# Addition of chemotherapy to radiotherapy promotes progenitor-exhausted CD8^+^ T-cell clonal dominance in head and neck cancer

**DOI:** 10.1158/2326-6066.CIR-26-0706

**Published:** 2026-10-12

**Authors:** Charleen Chan Wah Hak, Anton Patrikeev, Antonio Rullan, Emmanuel C Patin, Victoria Roulstone, Lisa C Hubbard, Matthew Guelbert, Elizabeth S Appleton, Shane Foo, Isaac Dean, Amy Burley, Joan N Kyula-Currie, Holly Baldock, Jen Y Lee, Pablo Nenclares, Hannah Nanapragasam, Carmen Murano, Malin Pedersen, Maggie Chon U Cheang, Shreerang A Bhide, Masahiro Ono, Kevin J Harrington, Alan A Melcher

**Affiliations:** 1 Division of Radiotherapy and Imaging, The Institute of Cancer Research, London SW3 6JB, UK; 2 Institute of Immunity and Transplantation, University College London; 3 Flow Cytometry Facility, The Institute of Cancer Research, London SW3 6JB, UK; 4 Biological Services Unit, The Institute of Cancer Research, London SW3 6JB, UK; 5 Head and Neck Unit, The Royal Marsden Hospital, London, UK; 6 Clinical Trials and Statistics Unit at The Institute of Cancer Research (ICR-CTSU); 7 Faculty of Natural Sciences, Department of Life Sciences, Imperial College London, UK

## Abstract

Concomitant chemoradiotherapy (CRT) is a standard-of-care treatment for unresectable locally advanced head and neck squamous cell carcinoma (LA-SCCHN), but its immune effects, particularly relative to radiotherapy (RT) alone, remain incompletely understood. Using syngeneic murine models, we integrated Nr4a3-Tocky reporter analysis with single-cell transcriptomics and T cell receptor clonotyping to comprehensively profile intratumoural CD8^+^ T-cell responses following RT and CRT. We show that CRT uniquely drives robust clonal expansion of antigen-engaged CD8^+^ T-cells and biases differentiation toward progenitor-exhausted (T_PEX_) states, whereas RT favours terminal exhaustion (T_EX_). Single-cell analyses further reveal preferential clonal dominance within T_PEX_ populations following CRT. In peripheral blood mononuclear cells from patients treated with CRT, T_EX_ populations predominated early after treatment, followed by delayed expansion of circulating T_PEX_-like populations at 3 months, suggesting phased recovery of progenitor-like T-cell states. Together, these findings demonstrate that CRT and RT induce distinct CD8^+^ T-cell differentiation trajectories and clonal architectures, providing insight into treatment-induced immune remodelling in LA-SCCHN.

## Introduction

The cancer-immunity cycle describes a dynamic process in which tumour cell death promotes antigen release, dendritic cell activation, and T-cell priming, ultimately driving cytotoxic immune responses against malignant cells (1). However, in the tumour microenvironment (TME), chronic antigen exposure and immunosuppressive signalling often impair T-cell differentiation, leading to dysfunctional states collectively termed T-cell exhaustion (2). Exhausted CD8^+^ T-cells are characterised by reduced effector function and sustained expression of inhibitory receptors such as PD-1, LAG-3, and TIM-3, limiting durable antitumour immunity (3). This state is generally seen as irreversible, particularly in the terminally exhausted (T_EX_) subset. However, the less differentiated precursor exhausted (T_PEX_) population, marked by the transcription factor TCF1, retains proliferative potential and responsiveness to immune checkpoint inhibitors (ICIs), positioning it as a key target for therapeutic approaches that seek to reinvigorate antitumour immunity (4–6).

While the spotlight in recent years has deservedly fallen on ICIs, an important paradigm shift has emerged: radiotherapy (RT) and chemoradiotherapy (CRT) – historically viewed as being immunosuppressive – can also profoundly modulate the immune landscape in an immunostimulatory fashion and enhance responses to immunotherapy (7). RT can induce immunogenic tumour cell death and enhance antigen release, potentially converting tumours into an *in-situ* vaccine capable of priming systemic, tumour-specific immune responses (8). Furthermore, recent studies have shown that CRT reshapes T-cell receptor (TCR) clonality by selectively expanding dominant T-cell clones associated with tumour specificity in head and neck, cervical, and rectal carcinomas (9–11). However, the functional consequences of this expansion remain unclear.

The incidence of squamous cell carcinoma of the head and neck (SCCHN) is rapidly rising in the developed world and it ranks as the seventh commonest cancer diagnosis worldwide (12). Definitive CRT remains standard-of-care for patients with unresected locally-advanced (LA) disease. Although the addition of cisplatin increases toxicity compared to RT alone, it improves local control and overall survival (13). The benefit of platin-based CRT is statistically significant and clinically meaningful, but relatively modest (approximately 6.5%) (13). Attempts to replace cisplatin with alternative agents or to de-escalate treatment intensity have not consistently improved outcomes (14, 15), reinforcing CRT as the therapeutic backbone in this setting. Despite substantial advances in refining locoregional therapy (16, 17), overall survival outcomes for patients with LA-SCCHN have plateaued, highlighting the need for strategies that move beyond optimisation of CRT parameters alone.

Efforts to integrate ICIs with RT or CRT have yielded disappointing results in several phase III trials (18–22), underscoring a gap in mechanistic understanding of how standard therapies influence immune responsiveness. Most preclinical studies have focused on RT alone, leaving the immunological consequences of CRT comparatively underexplored. In particular, it remains unclear how CRT versus RT differentially affects CD8^+^ T-cell differentiation trajectories, exhaustion states, and clonal dynamics within tumours. Antigen persistence during CRT may simultaneously promote clonal expansion and drive exhaustion in highly inflammatory environments like SCCHN (23), but whether this favours durable T_PEX_ maintenance or terminal dysfunction is unknown. Emerging evidence underscores the prognostic and functional relevance of different CD8^+^ T-cell exhaustion states in SCCHN, with both T_PEX_ and subsets of T_EX_ cells being associated with clinical benefit depending on context (23, 24). TCR signalling strength and antigen engagement have also been implicated as key determinants of responsiveness to immunotherapy (25). Together, these observations suggest that therapeutic modulation of exhaustion trajectories may be critical for improving responses to combined modality treatment.

In this study, we use syngeneic immunocompetent mouse models to dissect the immunological consequences of RT versus CRT in SCCHN. We demonstrate that although both modalities increase CD8^+^ T-cell infiltration, only CRT induces a CD8^+^ T-cell-dependent antitumour response. By integrating Nr4a3-Tocky reporter systems with single-cell transcriptomic and TCR clonotype analyses, we show that CRT promotes antigen-engaged CD8^+^ T-cell clonal expansion and biases differentiation toward a progenitor exhausted (T_PEX_) state rather than terminal exhaustion (T_EX_). Translational analyses of patient peripheral blood mononuclear cells (PBMCs) further reveal systemic immune modulation following CRT, including dynamic changes in exhaustion and progenitor-like CD8^+^ T-cell subsets. Together, these findings define distinct immune consequences of RT and CRT and identify differential regulation of CD8^+^ T-cell exhaustion states as a feature of CRT in SCCHN.

## Materials and Methods

### Mouse models of head and neck cancer

All procedures involving animals were approved by the Animal Ethics Committee at The Institute of Cancer Research in accordance with National Home Office Regulations under the Animals (Scientific Procedures) Act 1986. Wild-type (Charles River, RRID:MGI:2159769) or Nr4a3-Tocky C57BL/6 female mice (8-14 weeks) were housed under specific pathogen-free conditions. The HPV-positive syngeneic mouse SCCHN mEER cell line (RRID:CVCL_B6J2) was a gift from Dr Paola Vermeer (Sanford Research, USA) in 2018-2019 and HPV-negative C57BL/6 carcinogen-induced mouse oral cancer cell line MOC1 (26) (RRID:CVCL_ZD32) were kindly provided by Ravindra Uppaluri (Dana-Farber Cancer Institute) in 2019-2021. Cells were cultured in complete DMEM (Gibco, 41966029) supplemented with heat-inactivated 5/10% FBS (Gibco, A5256701) and in-house preparations of L-glutamine (1%) and penicillin-streptomycin (0.5%; ICR Media Laboratory). Cell lines used were passaged for approximately 5-10 passages before a new aliquot was thawed. Cell line authentication was performed with whole-exome sequencing. Potential mycoplasma contamination was routinely monitored using the MycoStrip Mycoplasma Detection Kit (InvivoGen, RRID:SCR_026997, rep-mysv2-10), and no infection was detected. 1 × 10^6^ mEER cells or 4 × 10^6^ MOC1 cells were injected subcutaneously into the right flank under isoflurane anaesthesia (IsoFlo, 100% w/w isoflurane; Zoetis UK Limited). Tumour and weight measurements were taken twice weekly by an independent animal technician who was blinded to therapeutic interventions. Tumour volume (mm^3^) was calculated as length × width × height × 0.5236. For all studies, mice were randomised prior to treatment initiation for comparable tumour volumes between cohorts. Mice were euthanised at predefined humane endpoints, including tumour diameter ≥15 mm, >18% body weight loss, or clinical signs of distress.

### *In vivo* treatments

*In vivo* treatments were commenced when tumour volumes reached approximately 30-60 mm^3^ (mEER: day 14–18; MOC1: day 28–35 post-implantation). For RT, mice were anaesthetised with ketamine hydrochloride (100 mg/kg; Ketavet, 100 mg/mL; Zoetis) and xylazine hydrochloride (16 mg/kg; Rompun, 2% w/v; Elanco UK AH Limited) and positioned prone under lead shielding with a 16 mm aperture over the tumour. mEER tumours received 8 Gy × 3 fractions and MOC1 tumours 6 Gy × 3 fractions on alternate days using an AGO 250 kV X-ray unit (dose rate: 1.62 Gy/min). Dosimetry was verified using a calibrated Farmer Chamber and Unidos-E Dosimeter (both PTW).

For CRT, cisplatin (7 mg/kg, Sigma Aldrich, P4394) was administered once only by intraperitoneal (i.p.) injection one hour prior to the first fraction of RT. CD8^+^ T-cell depletion was performed using anti-CD8α (loading dose 400 µg, maintenance 200 µg twice weekly; clone: 2.43, BioXCell; BE0061; RRID:AB_1125541) or rat IgG2b isotype control (clone: LTF-2, BioXCell; BE0090; RRID:AB_3662740) for up to 10 doses. Confirmation of *in vivo* CD8^+^ T-cell depletion using this regimen is outlined in Fig. S1. Anti-PD-1 (200 µg, clone: RMP1-14, BioXCell; BE0146; RRID:AB_10949053) or rat IgG2a isotype control (clone: 2A3, BioXCell; BE0089; RRID:AB_1107769) were administered i.p. twice-weekly for up to six doses.

To assess long-term protective antitumour immunity, mice that achieved durable complete tumour remission following primary RT or CRT were rechallenged 60 days after complete tumour regression. Age-matched treatment-naïve mice served as controls. All mice were rechallenged simultaneously by subcutaneous implantation of 1 × 10^6^ mEER cells into the contralateral flank using the protocol described above.

### Histopathology

Formalin-fixed paraffin-embedded mouse tissues were stained for CD8α by immunohistochemistry. Anti-CD8α (clone: EPR21769; Abcam; ab217344, RRID:AB_3095639) was used with heat-induced epitope retrieval using Agilent Target Retrieval solution pH9 and Agilent rabbit EnVision reagent as the detection system. Slides were scanned at 20× magnification using a Hamamatsu Nanozoomer (RRID:SCR_023762) and analysed in QuPath v0.5.1 (RRID:SCR_018257). Images were analysed as H-DAB brightfield images. Tumour regions were manually annotated, with areas of necrosis excluded. CD8α-positive cells were quantified using QuPath Positive Cell Detection, with detection parameters optimised for each tumour model based on haematoxylin and DAB staining and then applied consistently to all samples within that model. CD8α-positive cell density was expressed as positive cells per mm^2^ of viable tumour tissue.

### Flow cytometry analysis

Tumours were dissociated mechanically using scissors and enzymatically digested using collagenase type 76 I-S (Sigma-Aldrich, C1639), Dispase II (Sigma-Aldrich, D4693) and DNase I (Roche, 10104159001). Samples were then filtered through 70 μm strainers and washed with FACS buffer. Lymph nodes were mechanically dissociated and similarly filtered. For peripheral mouse blood, red blood cell lysis was performed by incubation in ACK lysis buffer (Thermo Fisher Scientific, A1049201) for 2 min. Human cryopreserved PBMCs were thawed using CTL Anti-Aggregate Wash Supplement (Cellular Technology Limited, CTL-AA-005) according to the manufacturer’s protocol. Samples were centrifuged at 300 × g for 5 min at 4°C, washed and resuspended in FACS buffer for Fc blocking and staining. Cells were stained following Fc receptor blockade, with anti-mouse CD16/CD32 Fc-receptor blocker (BD, 553142; RRID:AB_394657) or anti-human Fc-receptor blocker (BD, 564220; RRID:AB_2869554), using antibodies listed in Table S1-S2, with dead cells excluded using Fixable Viability Dye eFluor 780 (Thermo Fisher Scientific, 65-0865-14). Intracellular staining was performed using the Foxp3/Transcription Factor Staining Buffer Set (Thermo Fisher Scientific, 00-5523-00) as per manufacturer’s protocol. Samples were acquired on a BD FACSymphony A5 (RRID:SCR_022538) using single-stained samples or UltraComp eBeads compensation beads (Thermo Fisher Scientific, 01-2222-41) for compensation and CountBright Absolute Counting Beads (Thermo Fisher Scientific, C36950) for absolute cell quantification. Analysis was conducted using FlowJo v10 (RRID:SCR_008520), and Tocky timer analyses were performed using the TockyAnalysis R package in R v3.6.3. Gating strategies are shown in Fig. S2-S5.

### Single-cell RNA, TCR and AbSeq profiling

Tumours and TDLNs from mEER-bearing Nr4a3-Tocky mice were collected 10 days following the final dose of RT. Single-cell suspensions were pooled by treatment group and tissue type. Tumour samples were enriched for CD45^+^ cells with mouse CD45 (TIL) microbeads (Miltenyi Biotec, 130-052-301; RRID:AB_3224404) according to the manufacturer’s protocol, stained with AbSeq antibodies (Table S3) and sample tags, and sorted into Tocky-defined populations (“new,” “persistent,” “arrested,” and “timer-negative”) using a BD FACSymphony S6 cell sorter (Fig. S6; RRID:SCR_028281). Cells were processed using the BD Rhapsody platform (RRID:SCR_027096) for whole-transcriptome analysis (WTA), AbSeq, and TCR sequencing according to the manufacturer protocols. The following kits were used: BD Single-Cell Multiplexing Kit (BD, 633793), BD Rhapsody cDNA Kit (BD, 633773), BD Rhapsody WTA Amplification Kit (BD, 633801) and Mouse TCR/BCR Amplification Kit (BD, 666282). Libraries were sequenced on Illumina NovaSeq instruments.

Combined data from three WTA sequencing runs, including repeat sequencing due to technical issues, yielded RNA libraries with a mean depth of 8,016 reads and 2,644 molecules per cell. AbSeq libraries reached a mean depth of 8,552 reads and 3,098 molecules per cell, while TCR libraries achieved a mean depth of 2,396 reads and 6.35 molecules per cell. Raw data were aligned and pre-processed using the BD Rhapsody pipeline, then filtered to retain cells with ≥10 detected proteins, ≥500 AbSeq unique molecular identifiers (UMIs), and <20% mitochondrial content. Following quality control, at least 3,600 cells were retained in each of the “persistent”, “arrested”, and “timer-negative” populations, whereas approximately 200 “new” Tocky cells passed QC, limiting analyses within this subset. Paired scRNA-seq and TCR V(D)J data suitable for integrated clonotype analysis were available only for the “arrested” and “timer-negative” populations. Productive clonotypes were recovered from 78.2% (1,410/1,803) of arrested and 81.8% (1,084/1,325) of timer-negative T-cells and subsequently mapped onto UMAP-defined transcriptional subsets. Downstream analyses were performed in R v4.3.3.

### Bulk RNA and TCR sequencing

Mouse tumours were harvested 14 days after final radiation dose and snap frozen on dry ice. RNA was extracted from homogenised tissue using the RNeasy Plus Mini Kit (Qiagen, 74134) with QIAshredder (Qiagen, 79656) according to the manufacturer’s protocol. Library preparation and sequencing were performed using strand-specific polyA-selected libraries with 2×150 bp paired-end sequencing on an Illumina NovaSeq platform. Transcript-level abundance was quantified using Salmon v1.10.1 (RRID:SCR_017036) against the GRCm39 mouse reference (Ensembl release 103) and aggregated to gene level using tximport v1.34 R package (RRID:SCR_016752) (27). Differential expression analysis was performed using DESeq2 (RRID:SCR_000154) (28) (pseudogenes were excluded and only genes with more than 10 reads in at least 5 samples were kept in the analysis). Cell type signature scoring was performed using the GSVA package (v2.0.4) (29, 30), and gene set enrichment analysis was performed using the clusterProfiler package (v4.14.4; RRID:SCR_016884) (31).

### Human studies

Peripheral blood mononuclear cells (PBMCs) were collected from patients with LA-SCCHN treated with definitive RT (n=4) or CRT (n=8) as part of the INOVATE study (Investigation of Novel Plasma Human Papilloma Virus DNA Assay for Treatment Response Estimation in Head and Neck Cancer, ISRCTN32335415). The INOVATE study was approved by the London-Bloomsbury Research Ethics Committee (19/LO/1558). Written informed consent was obtained from all participants prior to study enrolment, and the study was conducted in accordance with the ethical principles of the Declaration of Helsinki. All patients were aged ≥18 years with newly diagnosed HPV-associated (p16-positive) T1-T2/N1-3 or T3-T4/N0-3 squamous cell carcinoma of the oropharynx undergoing radical treatment with RT or CRT. As INOVATE predominantly recruited patients receiving CRT, reflecting standard-of-care treatment for locally-advanced disease, all available RT-treated patients and a subset of CRT-treated patients with longitudinal PBMC samples were included in this exploratory analysis. Samples were collected at baseline, week 3 of RT (RTW3), and at 3 months post-treatment (RTFU); one patient in the RT cohort had a follow-up collection at 6 weeks due to scheduling constraints. PBMCs were isolated using a density gradient medium (Lymphoprep, STEMCELL Technologies, 18061) and stored in liquid nitrogen until analysis.

### Statistical analysis

Statistical analyses were performed using GraphPad Prism v10 (RRID:SCR_002798). Data are presented as means ± SEM and derive from single or pooled results of two to three independent experiments. Tumour growth was compared using area under the curve (AUC). Survival differences were assessed using the log-rank (Mantel-Cox) test. For group comparisons, parametric or non-parametric tests were used as appropriate based on normality (Shapiro-Wilk test), including unpaired t-test or Mann-Whitney U test for two-group comparisons, and one-way ANOVA with Tukey’s test or Kruskal-Wallis test with Dunn’s correction for multiple groups. Wilcoxon signed-rank test was used for paired fold-change analyses. Outliers were excluded using the ROUT test (Q=1%), and statistical significance was defined as p < 0.05.

## Results

### Radiotherapy (RT) and chemoradiotherapy (CRT) both increase CD8^+^ T-cell infiltration into tumours, but the therapeutic effect is CD8^+^ T-cell dependent only with CRT

**Fig. 1:**
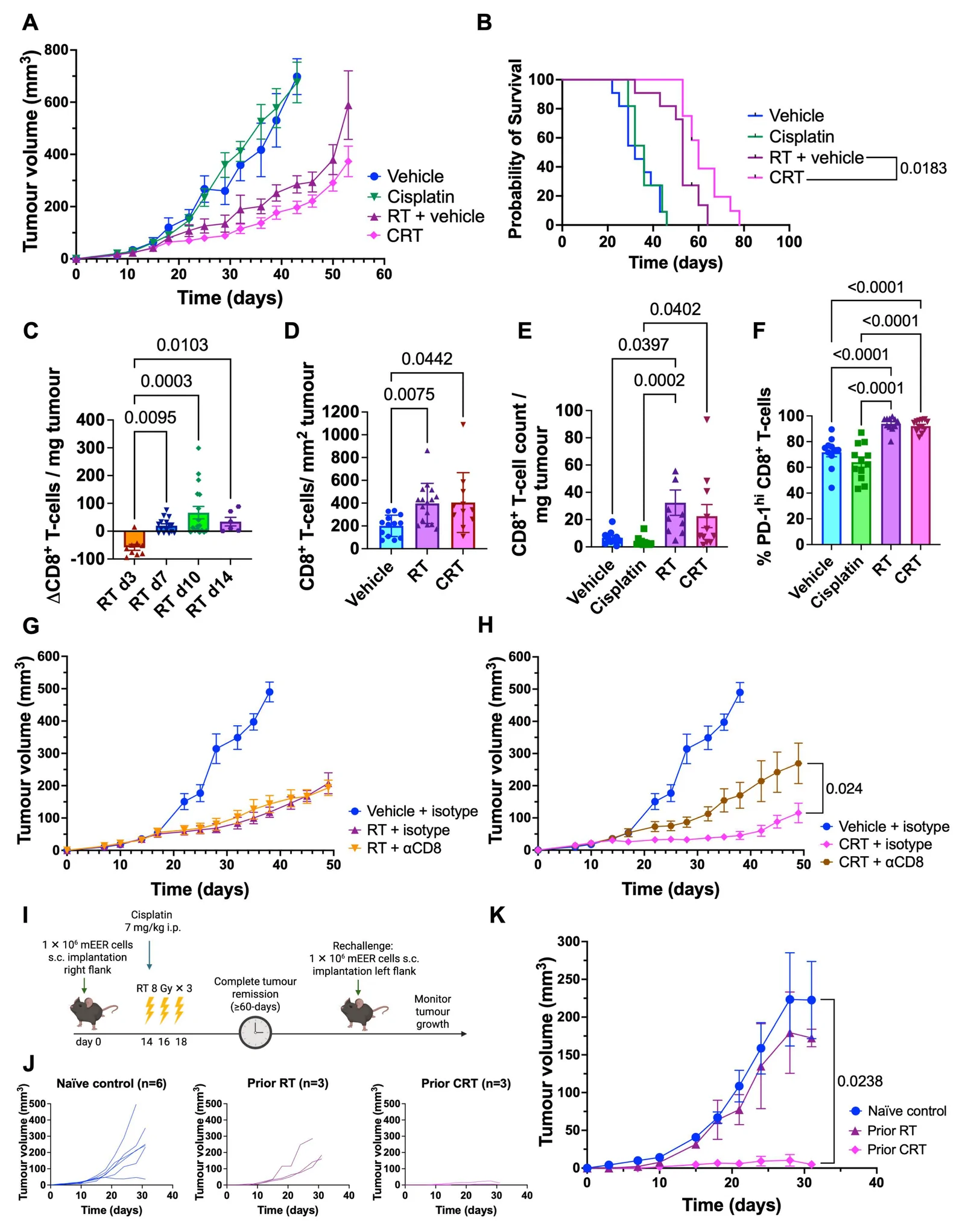
Both radiotherapy (RT) and chemoradiotherapy (CRT) increase CD8^+^ T-cell infiltration into tumours, but therapy is only CD8^+^ T-cell dependent in CRT. Experiments performed in the mEER model. **A**. Tumour growth curves across indicated treatment conditions. **B**. Survival curves across indicated treatment conditions (A-B: Vehicle n=11, Cisplatin n=11, RT + vehicle n=11, CRT n=12; 2 independent experiments). **C**. Absolute change of CD8^+^ T-cell counts/mg tumour vs average of time-matched controls across indicated timepoints post-RT as measured by flow cytometry (RT d3 n = 9, RT d7 n = 15, RT d10 n = 17, RT d14 n = 6; 6 independent experiments of 2 consecutive timepoints). **D**. CD8^+^ T-cells/mm^2^ tumour at 14 days post-RT by immunohistochemistry, necrotic regions excluded, quantified by QuPath v. 0.3.0. (Vehicle n=13, RT n=16, CRT n=11; 3 independent experiments). **E**. CD8^+^ T-cell count/mg of tumour by flow cytometry (Vehicle n=10, Cisplatin n=11, RT n=10, CRT n=11; 2 independent experiments). **F**. Percentage of PD-1^hi^ CD8^+^ T-cells by flow cytometry (Vehicle n=11, Cisplatin n=12, RT n=11, CRT n=12; 2 independent experiments). **G-H:** Tumour growth curves of αCD8 depletion experiments, with mice receiving either RT **(G)** or CRT **(H)** (Vehicle + isotype n=6, RT + isotype n=6, RT + αCD8 n = 5, CRT + isotype n = 6, CRT + αCD8 n = 6; data from 1 experiment). **I**. Schematic of the tumour rechallenge experiment. Of the mice treated in the primary efficacy study, 3/12 RT-treated mice and 3/10 CRT-treated mice achieved durable complete tumour remission and were rechallenged 60 days after complete tumour regression by contralateral subcutaneous implantation of mEER cells. Age-matched treatment-naïve mice served as controls. *Created in BioRender. Harrington, K. (2026)* https://BioRender.com/fi2x76l. **J**. Individual tumour growth curves following tumour rechallenge. **K**. Mean tumour volume following tumour rechallenge (treatment-naïve, n=6; prior RT complete responders, n=3; prior CRT complete responders, n=3; 1 experiment). Results are presented as mean ± SEM and n denotes mice per group. Parametric statistics were applied only to normally distributed data (Shapiro-Wilk test). P values shown were determined by the log-rank (Mantel-Cox) test (B), Kruskal-Wallis test with Dunn’s multiple comparisons test (C-E), ordinary one-way ANOVA with Tukey’s multiple comparisons test (F), two-tailed unpaired t-test of tumour area under the curve (days 0-42) (H), and Mann-Whitney U test of tumour area under the curve (days 0-28) (K), *p*<0.05 was considered statistically significant.

Compared to RT, CRT yielded a modest, non-significant improvement in tumour control in the mEER preclinical model (Fig. 1A, S7A), in line with the relatively modest benefits reported in clinical meta-analysis (13). Survival was significantly prolonged in the mEER model (Fig. 1B). CD8^+^ tumour-infiltrating lymphocyte (TIL) dynamics varied over time following RT as determined by flow cytometry from baseline: numbers declined at day 3 post-RT, consistent with radiation-induced intratumoural lymphopenia, recovered by day 7 post-RT, and peaked at day 10 (Fig. 1C). Both RT and CRT increased CD8^+^ TIL abundance compared to vehicle or cisplatin-alone controls; however, no significant differences were observed between RT and CRT at day 10 or day 14 following treatment when quantified by immunohistochemistry (Fig. 1D) and flow cytometry (Fig. 1E). Furthermore, there were no significant differences in the percentage of PD-1^hi^ expressing CD8^+^ T-cells between RT and CRT treatment groups (Fig. 1F).

To assess the function of CD8^+^ T-cells, we performed depletion studies under RT and CRT conditions. In the mEER model, RT-mediated tumour control was CD8^+^ T-cell-independent (Fig. 1G, Fig. S7B), whereas CRT (cisplatin + RT) showed CD8^+^ T-cell-dependence, as depletion significantly decreased tumour control (Fig. 1H, Fig. S7B). To determine whether this CD8^+^ T-cell-dependent response generated durable protective immunity, mice that achieved complete tumour remission following primary treatment were rechallenged with mEER cells in the contralateral flank 60 days after tumour regression (Fig. 1I). Whereas tumours readily became established in treatment-naïve mice and in mice previously treated with RT, tumour growth was markedly restricted in mice previously treated with CRT (Fig. 1J-K), consistent with enhanced long-term systemic antitumour immunity following CRT. These findings indicate a mechanistic role for CD8^+^ T-cells in CRT response in this model. In the MOC1 model, CRT similarly yielded a modest, non-significant improvement in tumour control compared with RT (Fig. S8A-B), without a significant survival benefit (Fig. S8C). In this model, which exhibits higher baseline CD8^+^ T-cell infiltration, depletion affected neither RT nor CRT (Fig. S8D-F). Subsequent mechanistic experiments therefore focused on the mEER model, where CRT conferred a modest survival benefit in line with the clinical meta-analysis (13), exhibited CD8^+^ T-cell-dependent tumour control, and generated enhanced protection against tumour rechallenge. We next investigated whether these functional differences reflected distinct patterns of CD8^+^ T-cell antigen engagement.

### CRT reduces the proportion of persistently engaged CD8^+^ TILs compared to RT

Immunoprofiling via flow cytometry of mEER tumours showed that RT and CRT broadly enhanced lymphocyte activation, proliferation, and cytotoxicity, with CRT exerting the stronger effect overall (Fig. S9). Both treatments increased CD44 expression (Fig. S9A) and expanded granzyme B^+^/perforin^+^ cytotoxic populations (Fig. S9B) across CD8^+^ T-cell, NK, and NKT subsets. CRT significantly increased Ki67^+^ proliferating CD8^+^ T and NK cells (Fig. S9C), as well as T-bet^+^ (Fig. S9D) expression in Tconv and CD8^+^ T-cells. Effector memory (Fig. S9E) and PD-1^+^/TIM-3^+^ checkpoint-positive (Fig. S9F) CD8^+^ T-cells were also increased by both treatments.

**Fig. 2:**
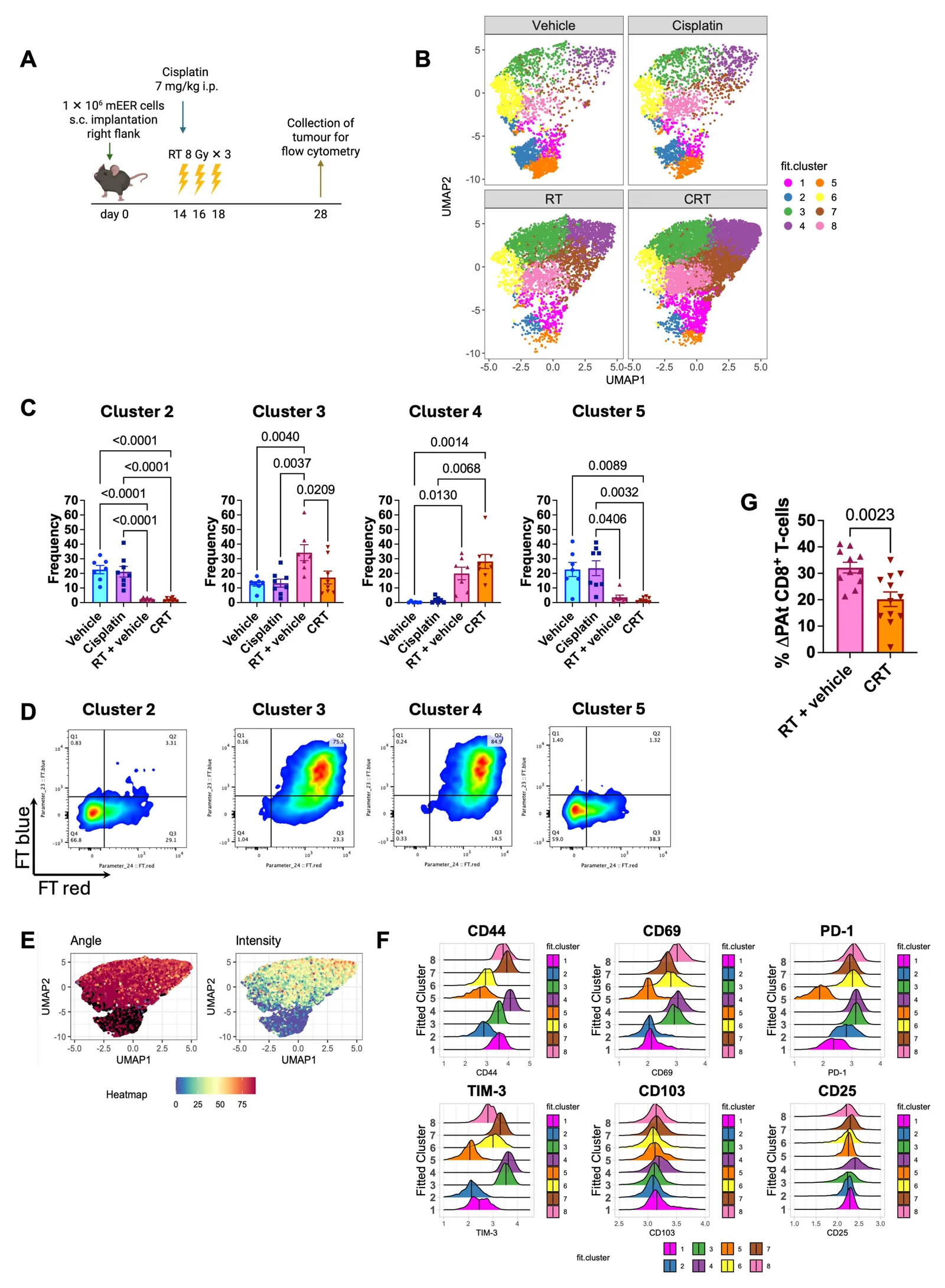
T-cell dynamics reveals differences in tumour T-cell populations between RT and CRT, with a reduced percentage increase in persistently engaged CD8^+^ T-cells in CRT versus RT. **A**. Schematic of the treatment timeline in Nr4a3-Tocky mEER tumour-bearing mice. *Created in BioRender. Harrington, K. (2026)* https://BioRender.com/fi2x76l. **B-F:** UMAP analysis of CD8^+^ tumour-infiltrating lymphocytes (TILs), results concatenated from 30 mice (Vehicle n=7, Cisplatin n=8, RT + vehicle n=7, CRT n=8; 1 experiment). **(B)** UMAP plot showing distribution of clusters 1–8 across treatment conditions. **(C)** Selected clusters 2, 3, 4 and 5 showing significant differences between treatment groups (Vehicle n=7, Cisplatin n=8, RT + vehicle n=7, CRT n=8). **(D)** Timer fluorescence patterns within clusters 2, 3, 4 and 5. Cluster 3 is defined by a persistent-arrested (PAt) Tocky profile with high activation and immune checkpoint expression. **(E)** Heatmap overlays of Tocky “angle” (antigen engagement) and “intensity” (TCR signalling strength). **(F)** Histograms illustrating marker expression by cluster. **G**. Percentage change in PAt CD8^+^ T-cells (% ΔPAt) in RT + vehicle and CRT groups versus the average of the vehicle group (RT + vehicle n=11, CRT n=12; 2 independent experiments). Results are means ± SEM and *n* denotes mice per group. Parametric statistics were only applied to normally distributed data (Shapiro-Wilk test). P-values shown were determined by ordinary one-way ANOVA with Tukey’s multiple comparisons test (C: clusters 2-3), Kruskal-Wallis test with Dunn’s multiple comparisons test (C: clusters 4-5) and Two-tailed Unpaired t test (G). Significant outliers were removed via ROUT test (Q = 1%), *p*<0.05 was considered statistically significant.

As cisplatin-based CRT was CD8^+^ T-cell-dependent, whereas RT alone was not, we next asked whether CRT differentially affects the quality or kinetics of T-cell antigen engagement (Fig. 2A). To address this, we employed the Nr4a3-Tocky (“Timer of cell kinetics and activity” reporter system) (32–34). This system utilises a Fluorescent Timer (FT) protein that undergoes a spontaneous, time-dependent shift in Timer fluorescence following TCR-mediated induction of Nr4a3, a TCR downstream nuclear receptor gene. The FT protein is initially expressed as an immature blue fluorophore, which matures into a stable red fluorophore with a half-life of approximately 4 hours (33). Consequently, newly antigen engaged T-cells express blue fluorescence (“new”), persistently engaged T-cells express both blue and red (“persistent”), and cells in which TCR signalling has ceased (“arrested”) express red fluorescence only. These dynamics are quantified using the ‘Timer Angle’, a trigonometric transformation where 0° (blue only) represents the onset of activation, 45° (blue and red) indicates sustained persistent signaling, and 90° (red only) identifies the cessation of TCR engagement. Additionally, the overall fluorescence intensity provides a qualitative measure of cumulative TCR signal strength.

To evaluate treatment-induced remodeling of the immune landscape, UMAP analysis was performed on concatenated CD8^+^ TIL data from all treatment groups (Fig. 2B). Both treatments increased the abundance of antigen-engaged, activated (CD44^hi^ CD69^hi^ CD25^hi^) and immune checkpoint-expressing (PD-1^hi^ TIM-3^hi^) CD8^+^ TILs compared to vehicle or cisplatin alone (Fig. 2C-F). This change was reflected in a significant increase in the frequency of cluster 4 (Fig. 2C), which was predominantly composed of cells exhibiting high Timer Intensity and ‘persistent’ TCR signal (Fig. 2D). Conversely, there was a corresponding decrease in the frequency of clusters 2 and 5 (Fig. 2C), which were dominated by a profile of timer-negative status, low activation and low immune checkpoint expression (Fig. 2D-F).

Focusing on the comparison between RT and CRT, a notable difference emerged within cluster 3. CRT-treated tumours exhibited a significant decrease in this cluster (Fig. 2C), which was defined by a “persistent-arrested” antigen engagement profile – characterised by sustained TCR signalling – despite displaying activation and immune checkpoint marker expression comparable to cluster 4 (Fig. 2D-F). Overall, CRT did not enhance overall antigen engagement compared to RT but, instead, specifically reduced the proportion of “persistent-arrested” (PAt) CD8^+^ TILs relative to vehicle controls (Fig. 2G). This subset may represent terminally exhausted cells, secondary to continuous antigen engagement (3, 35, 36).

Addition of anti-PD-1 (αPD-1) had no effect with RT alone, while there was a modest, but statistically non-significant, improvement in tumour control with αPD-1 in CRT-treated mice (Fig. S10). These findings suggest differential impacts of RT and CRT on T-cell exhaustion, motivating further interrogation of the transcriptional and phenotypic states of antigen-engaged TILs using single-cell approaches.

### CRT increases antigen-engaged TILs with effector memory (T_EM_) relative to naïve phenotype (T_N_), when compared to RT alone

**Fig. 3:**
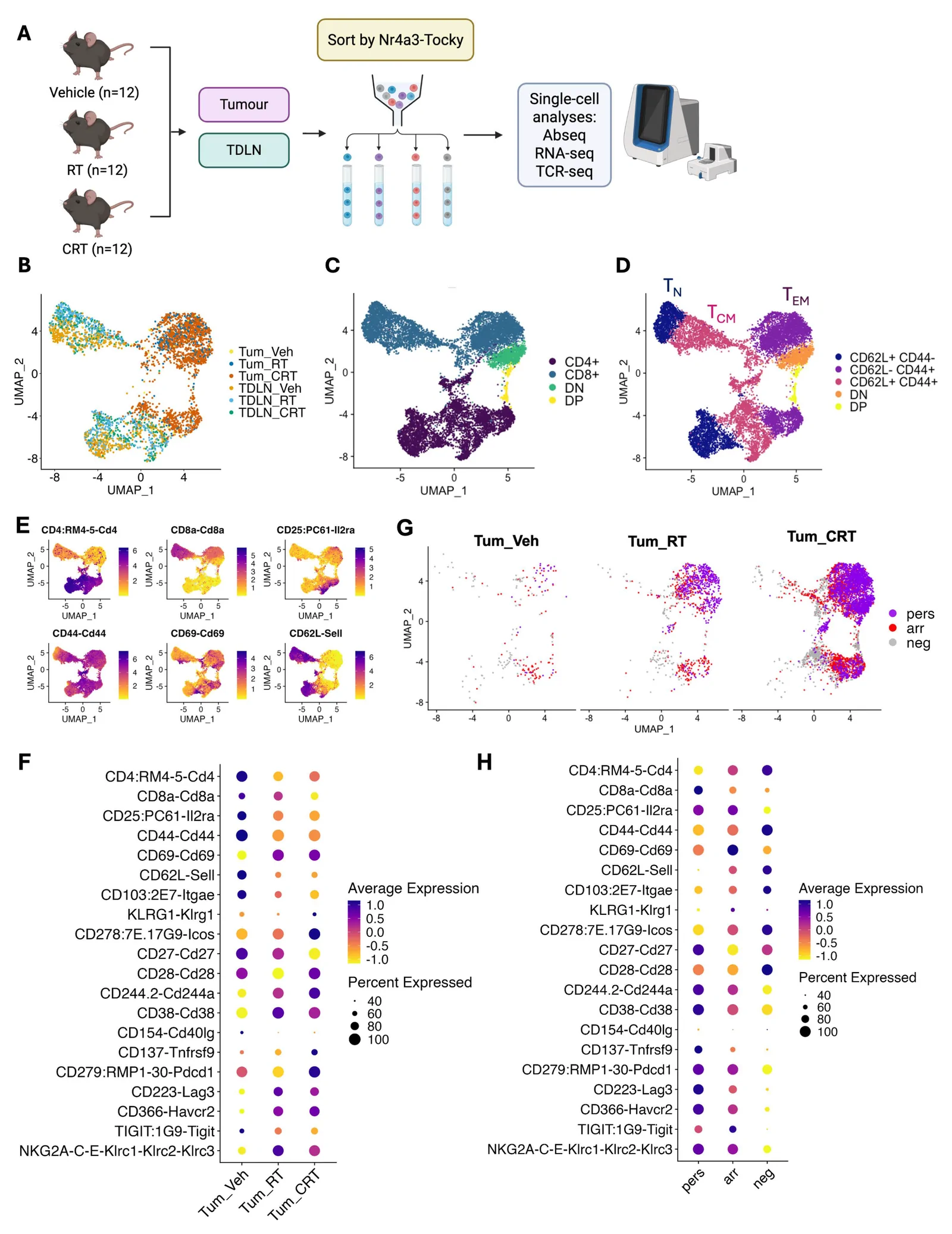
CRT increases antigen-engaged TILs with effector memory over naïve phenotype compared to RT; persistently engaged T-cells show higher inhibitory checkpoint expression linked to exhaustion. **A**. Experimental schema: Single-cell analysis was performed on tumour-infiltrating lymphocytes (TILs) from tumours (Tum) or TDLN harvested from mEER-bearing Nr4a3-Tocky mice treated with vehicle (Veh), RT or CRT. Cells were sorted according to Tocky antigen-engagement profile prior to integrated single-cell analyses and pooled by treatment group and tissue type (Vehicle n=12, RT n=12, CRT n=12 mice; 1 experiment). *Created in BioRender. Harrington, K. (2026)* https://BioRender.com/5yidrt9. **B-H:** Cells were subject to UMAP clustering based on Abseq expression profile. **(B)** UMAP by sample tag. **(C)** Cell annotation of T-cell subtype: CD4, CD8, DN (double negative) and DP (double positive). **(D)** Functional T-cell subtype annotation based on expression markers: T_N_ (naïve, CD62L^+^), TCM (central memory, CD62L^+^, CD44^+^), T_EM_ (effector memory, CD62L^-^ CD44^+^). **(E)** Heatmap of selected Abseq marker expression overlaid on UMAP. **(F)** Dot plot of Abseq marker expression in TILs by treatment. **(G)** Distribution of Tocky populations: “persistent” (pers), “arrested” (arr) and “timer negative” (neg) within the UMAP space. **(H)** Dot plots of Abseq marker expression across different Tocky populations in TILs.

To investigate the impact of CRT on T-cell phenotypes in more detail, we combined the Nr4a3-Tocky system with single-cell transcriptomic and protein profiling, using AbSeq, and TCR sequencing (Fig. 3A). Tumours and tumour-draining lymph nodes (TDLNs) were harvested from mice treated with vehicle, RT, or CRT, and cells were analysed by UMAP clustering based on AbSeq expression profiles (Table S3). Within the tumour, TILs were dominated by effector memory (T_EM_) phenotype (CD62L^-^ CD44^+^), whereas T-cells from TDLNs were primarily naïve (T_N_; CD62L^+^) and central memory (T_CM_; CD62L^+^, CD44^+^) (Fig. 3B-E).

Within TILs, following both RT and CRT, there was increased expression of activation markers (CD69, CD38), co-stimulatory receptors (CD278 [ICOS], CD137 [4-1BB]) and markers of inhibition/exhaustion (CD223 [LAG3], CD366 [TIM-3]), TIGIT and NKG2A-C-E) compared to vehicle. There was also a reduction in the average expression of the activation/Treg marker, CD25 compared to vehicle (Fig. 3F). Again, focusing particularly on differences with the addition of chemotherapy to RT, compared to RT alone, CRT showed increased KLRG1, CD278 (ICOS), CD28 and CD244a expression and reduced CD27 and CD38 expression. This profile is consistent with chemotherapy enhancing the induction of T_EM_ relative to naïve T-cells, compared to RT alone.

To further characterise PD-1^+^ and PD-1^−^ memory-like populations, T_CM_- and T_EM_-like CD8^+^ T-cells were stratified according to PD-1 expression, with additional assessment of the inhibitory receptors LAG-3, TIM-3 and TIGIT within these subsets (Fig. S11).

### Persistently-engaged T-cells express higher levels of multiple inhibitory checkpoint molecules consistent with continual antigen engagement driving T-cells towards exhaustion

To assess the temporal dynamics of antigen engagement across treatment groups, we mapped Tocky-defined populations (“persistent” [pers], “arrested” [arr], and “timer-negative” [neg]) onto the UMAP landscape (Fig. 3G). Untreated tumours (Tum_Veh) exhibited sparse antigen-engaged T-cells, with a predominance of timer-negative cells. In contrast, both RT and CRT markedly induced a striking predominance of antigen-engaged TILs within the TME. This expansion was most pronounced following CRT, which demonstrated a dense and cohesive accumulation of “persistent” (pers) T-cells (purple in Fig. 3G), indicative of sustained TCR signalling.

Examination of marker expression across Tocky populations showed that “persistent” T-cells expressed markedly higher levels of multiple immune checkpoint markers: CD279 (PD-1), CD233 (LAG-3), CD366 (TIM-3) and TIGIT, and the exhaustion-associated markers, CD244a and NKG2A-C-E, compared with other Tocky loci (Fig. 3H). This profile is consistent with continual antigen engagement driving T-cells towards an exhausted phenotype (3). In contrast, “timer-negative” T-cells demonstrated relatively higher expression of CD62L, CD103, CD27 and CD28, indicative of a less antigen-experienced or memory-associated state (Fig. 3H). While the “arrested” Tocky population displayed intermediate levels of immune checkpoints (e.g. PD-1, LAG-3, TIM-3), they reached a positive peak in CD69 expression and a negative peak in CD27. Furthermore, the inclusion of KLRG1-high cells within the arrested population suggests that these cells sustain enhanced cytotoxic potential (Fig. 3H). These dynamics suggest that T-cells with historical antigen engagement undergo an active remodelling phase at the “arrested” stage, characterised by reduced expression of multiple inhibitory checkpoint molecules and increased KLRG1 expression relative to “persistent” cells. While the single-cell transcriptomic analysis demonstrates increased overall antigen engagement following CRT, the persistent-arrested (PAt) subset defined by Nr4a3-Tocky dynamics cannot be directly identified within the scRNA-seq dataset (Fig. 3G). However, flow cytometry using the Tocky reporter revealed that CRT reduced the proportion of PAt cells compared with RT alone (Fig. 2G). Together, these findings indicate that CRT is associated with increased overall T-cell antigen engagement while reducing the accumulation of more exhausted PAt cells, relative to RT alone.

### CRT leads to more focused, clonally expanded CD8^+^ T-cell populations with an effector profile

**Fig. 4:**
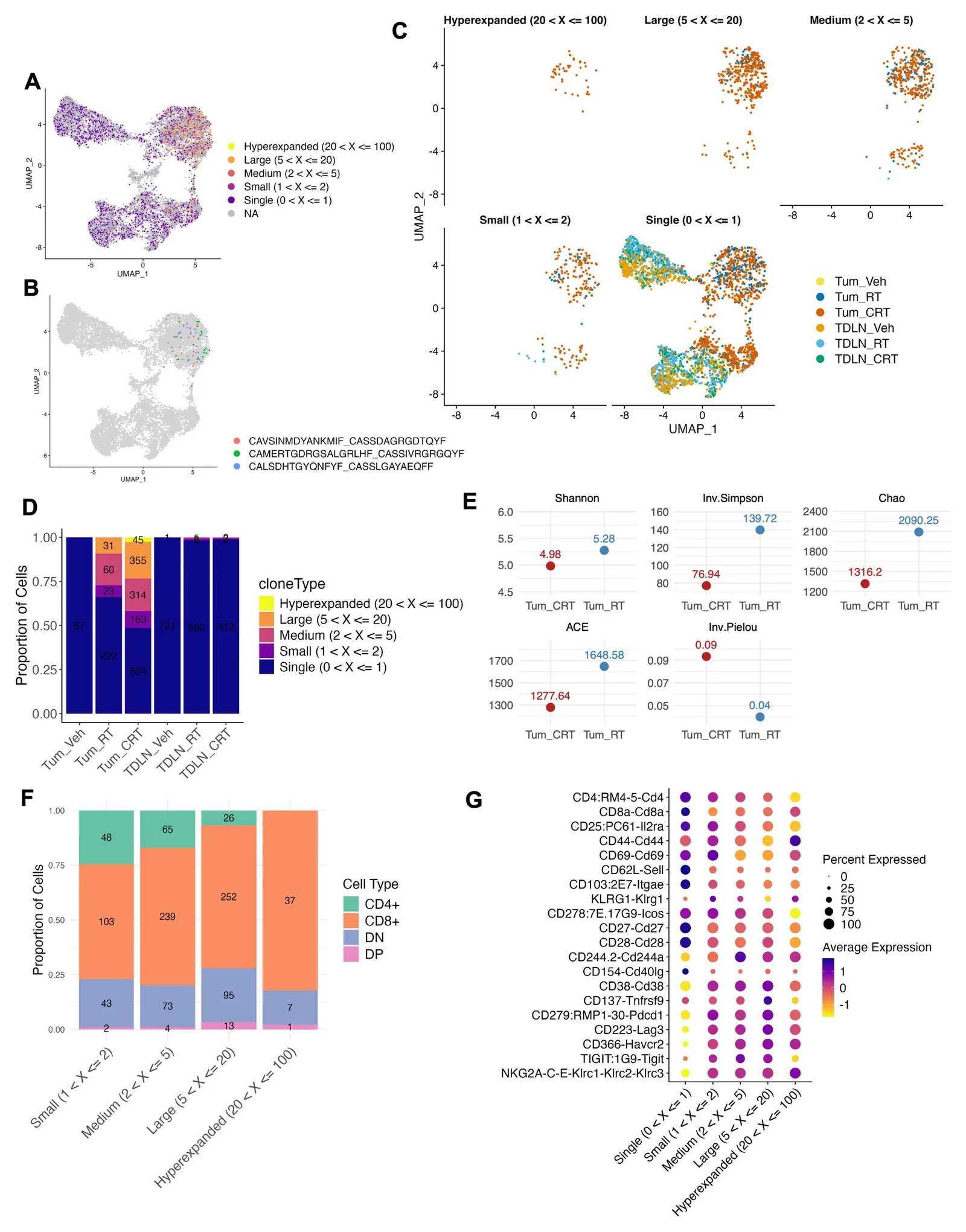
CRT leads to more focused, expanded CD8+ T-cell populations with an effector profile than RT. Single-cell TCR sequencing was performed on sorted tumour-infiltrating lymphocytes (TILs) from tumours (Tum) or TDLN harvested from mEER-bearing mice treated with vehicle (Veh), RT or CRT and pooled by treatment group and tissue type (Vehicle n=12, RT n=12, CRT n=12 mice, 1 experiment). **A**. Distribution of T-cell clonal expansion within UMAP space, categorised by clone frequency within the total TCR repertoire: hyperexpanded (>20 and ≤100), large (>5 and ≤20), medium (>2 and ≤5), small (>1 and ≤2) and single (>0 and ≤1). Cells without information on paired TCR α (alpha) and β (beta) chain CDR3 (Complementarity-Determining Region 3) sequence information are denoted by “NA”. **B**. Three CDR3 sequences which were hyperexpanded. **C**. Distribution of categories of clonal expansion within UMAP space by sample type and treatment group. **D**. Categories of clonal expansion by sample type and treatment group by proportion of cells **E**. Clonal diversity dotplots comparing various diversity indices between Tum_RT and Tum_CRT: Shannon; Inverse (Inv) Simpson; Chao1 (Chao) Abundance-based Coverage Estimator (ACE) and Inv Pielou. **F**. Proportions of T-cell type (CD4+, CD8+, double negative [DN], double positive [DP]) by categories of clonal expansion. **G**. Abseq expression marker profile across different clonal expansion categories.

Clonal expansion occurred almost exclusively within tumours treated with RT and CRT, with CRT leading to greater clonal expansion than RT alone (Fig. 4A-D). Hyperexpanded clones were made up of only three paired TCR α/β CDR3 sequences, that occurred exclusively in T-cells from tumours treated with CRT, highlighting striking clonal dominance (Fig. 4B). Following downsampling to the minimum clonotype count for comparability, consistently lower diversity in TILs from CRT- versus RT-treated tumours was still found by various indices (Fig. 4E). For reference, Shannon and Simpson indices combine clonotype richness and evenness, Chao1 and ACE indices estimate unseen diversity and the Inverse Pielou index was used to assess clonal skewing (37). Hence, the TCR repertoire in the CRT group was less even than RT and more clonal/skewed towards dominant clones. With increasing clonal expansion, a progressive predominance of CD8^+^ over CD4^+^ T-cells was observed, with hyperexpansion occurring exclusively within the CD8^+^ compartment (Fig. 4F).

Hence, CRT reshaped the TCR repertoire of TILs and altered the expression profile of hyperexpanded CD8^+^ T-cells. In all singleton clones across treatment conditions, there was a predominance of naïve or early memory (CD44^lo^, CD62L^hi^, CD27^hi^, CD28^hi^) and/or tissue-resident T-cells (CD103^hi^) (Fig. 4G). When the expression profile of the hyperexpanded clones (seen only with CRT) was examined, relatively higher expression of the activation marker CD44 was observed, with relatively lower multiple immune-checkpoint /inhibitory markers – CD279 (PD-1), CD223 (LAG-3), TIGIT and CD38, compared to the other clonal subgroups (Fig. 4G). These findings suggest that CRT-induced hyperexpanded CD8^+^ T-cells predominately acquire an effector-like, rather than exhausted, phenotype.

### CRT leads to greater clonal expansion of antigen-engaged CD8^+^ T-cells than RT

**Fig. 5:**
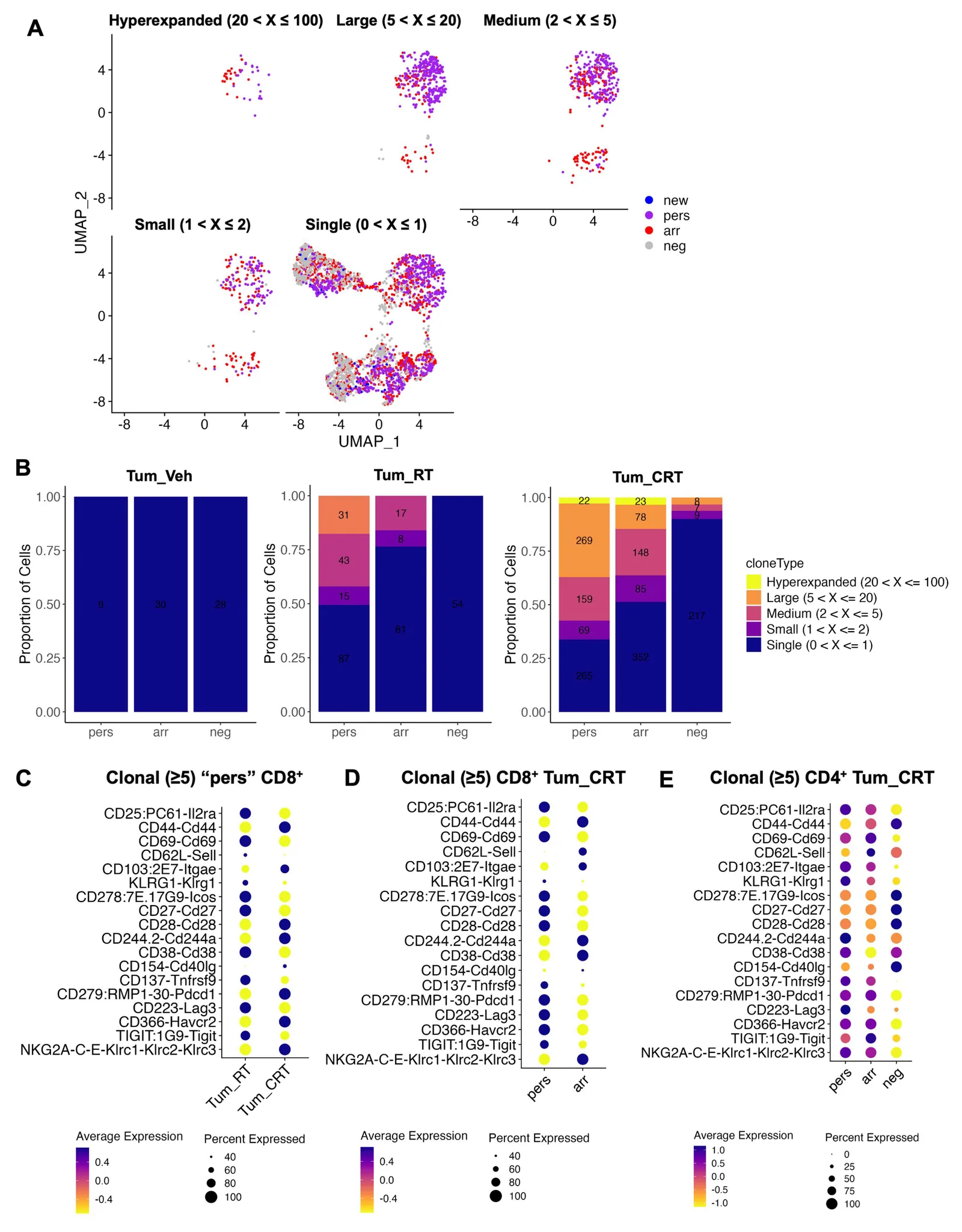
CRT leads to greater clonal expansion of antigen-engaged T-cells than RT, and clonal CD8 TILs treated with CRT show relative acquisition of a less exhausted phenotype on transition from persistent (“pers”), to historical, T-cell engagement (“arr”). Single-cell analysis was performed on sorted tumour-infiltrating lymphocytes (TILs) from tumours (Tum) or TDLN harvested from mEER-bearing mice treated with vehicle (Veh), RT or CRT and pooled by treatment group and tissue type (Vehicle n=12, RT n=12, CRT n=12 mice, 1 experiment). **A**. Distribution of Tocky populations within UMAP space according to categories of clonal expansion: “new”, “persistent” (pers), “arrested” (arr) and “timer negative” (neg). **B**. Proportions of cells undergoing clonal expansion by Tocky population. **C-E:** Abseq marker expression profile of various TILs subsets: **(C)** Clonal (≥ 5) and “persistent” CD8^+^ TILs by treatment type, **(D)** Clonal (≥ 5) CD8^+^ TILs from mice treated with CRT (Tum_CRT) by Tocky population and **(E)** Clonal (≥ 5) CD4^+^ TILs treated with CRT (Tum_CRT) by Tocky population.

Correlation of TCR-seq data with the Tocky profile revealed that CRT induced greater clonal expansion of antigen-engaged tumour-infiltrating CD8^+^ T-cells (“persistent” and “arrested”) compared to RT, whereas vehicle-treated tumours showed minimal clonal expansion (Fig. 5A-B). In contrast, TILs lacking evidence of antigen engagement at the time of analysis (“timer-negative”) demonstrated limited clonal expansion across treatment groups.

Given that Timer fluorescence reflects real-time TCR signalling dynamics, the enrichment of expanded clonotypes within the “persistent” compartment in CRT-treated tumours (Fig. 5B) suggests that a discrete subset of clones is either undergoing sustained proliferative expansion driven by continuous antigen stimulation or, alternatively, that once expanded, these clones preferentially occupy and remain within the persistently engaged niche.

Despite treatment-matched sampling, we observed limited clonal overlap between tumour and TDLN compartments (Fig. S12). Together with the apparent confinement of expanded clonotypes to defined Tocky signalling stages (Fig. 5B, S13), this supports a model in which CRT promotes spatially restricted, antigen-dependent clonal expansion within the TME.

### Clonal CD8^+^ TILs treated with CRT show relative acquisition of a less exhausted phenotype as they progress from “persistent” to “arrested”

To capture broader antigen-driven activity, the expression profile of specific clonal populations (frequency ≥5) was studied in relation to their Tocky profile in different samples. Firstly, clonal “persistent” CD8^+^ TILs were compared between tumours treated with RT and CRT; CD8^+^ TILs from CRT-treated tumours showed increased expression of activation and co-stimulation markers (CD44, CD28 and CD154 [CD40L]), the residency marker CD103, and antigen-experienced/ exhaustion-associated markers (CD244a, PD-1, CD366 [TIM-3], NKG2A-C-E), as compared to RT-treated tumours (Fig. 5C). Overall, this suggests that continuously antigen-engaged CD8^+^ TILs shift towards a more activated, tissue-resident, and exhaustion-associated phenotype following CRT, rather than RT.

Next, all clonal CD8^+^ TILs with a frequency ≥5 from CRT-treated tumours were examined by Tocky profile; these were “persistent” versus “arrested”, as there were no sufficiently expanded cells in the “new” or “timer negative” subsets (Fig. 5D). Here again, consistent with the model of chronic antigen stimulation driving exhaustion, persistently antigen-engaged CD8^+^ TILs exhibited reduced activation (CD44) and increased expression of multiple immune checkpoints, CD279 (PD-1), CD223 (LAG-3), CD366 (TIM-3) and TIGIT, compared to historically antigen-engaged, but now “arrested”, cells. Moreover, a small percentage of “persistent” cells showed elevated levels of the terminal differentiation marker KLRG1 compared to “arrested”. Collectively, these findings suggest that progression along the Tocky trajectory from “persistent” to “arrested”, is associated with reduced expression of multiple inhibitory checkpoint molecules and acquisition of a less exhaustion-associated phenotypic profile. However, the relationship between Tocky state and canonical T-cell differentiation states cannot be determined from the present dataset. Of note, this analysis could not be performed for TILs from RT-treated tumours, as there were no “arrested” clonal (frequency ≥5) CD8^+^ TILs present in this subset, only “persistent” cells.

Clonal CD4^+^ TILs, with a frequency ≥5, were only present in tumours treated with CRT and not RT (Fig. 5E). This is consistent with a model in which CD4^+^ T-cells support enhanced clonal expansion of CD8^+^ TILs following CRT more effectively than after RT alone. Similarities were observed in the expression profiles of “persistent” and “arrested” clonal CD4^+^ and CD8^+^ TILs; for example, “persistent” CD4^+^ TILs also exhibited higher expression of inhibitory/exhaustion-associated markers such as LAG3 and KLRG1, as well as activation markers CD25 and CD137 (4-1BB), compared to their “arrested” counterparts (Fig 5D-E). These are consistent with “persistent” cells representing a more chronically antigen-engaged state with increased expression of exhaustion-associated markers in both CD4^+^ and CD8^+^ TILs.

### CRT compared to RT increases the proportion and clonal expansion of precursor exhausted (T_PEX_), relative to terminally exhausted (T_EX_), CD8^+^ TILs in the arrested phase

To define treatment-induced changes in T-cell states, sorted single T-cells were subjected to UMAP clustering based on their global transcriptomic profiles. scRNAseq data was only available for “arrested” (arr) and “timer negative” (neg) samples due to technical and QC limitations (see Supplementary Methods). Nevertheless, these samples represent the two extremes of the Timer expression trajectory. UMAP clustering initially identified 11 transcriptionally distinct T-cell clusters. Based on shared lineage markers, characteristic gene expression of T_PEX_ and T_EX_ states in the published literature (4, 6, 38), and proximity in low-dimensional space, closely related clusters were consolidated into 9 annotated T-cell subsets.

**Fig. 6:**
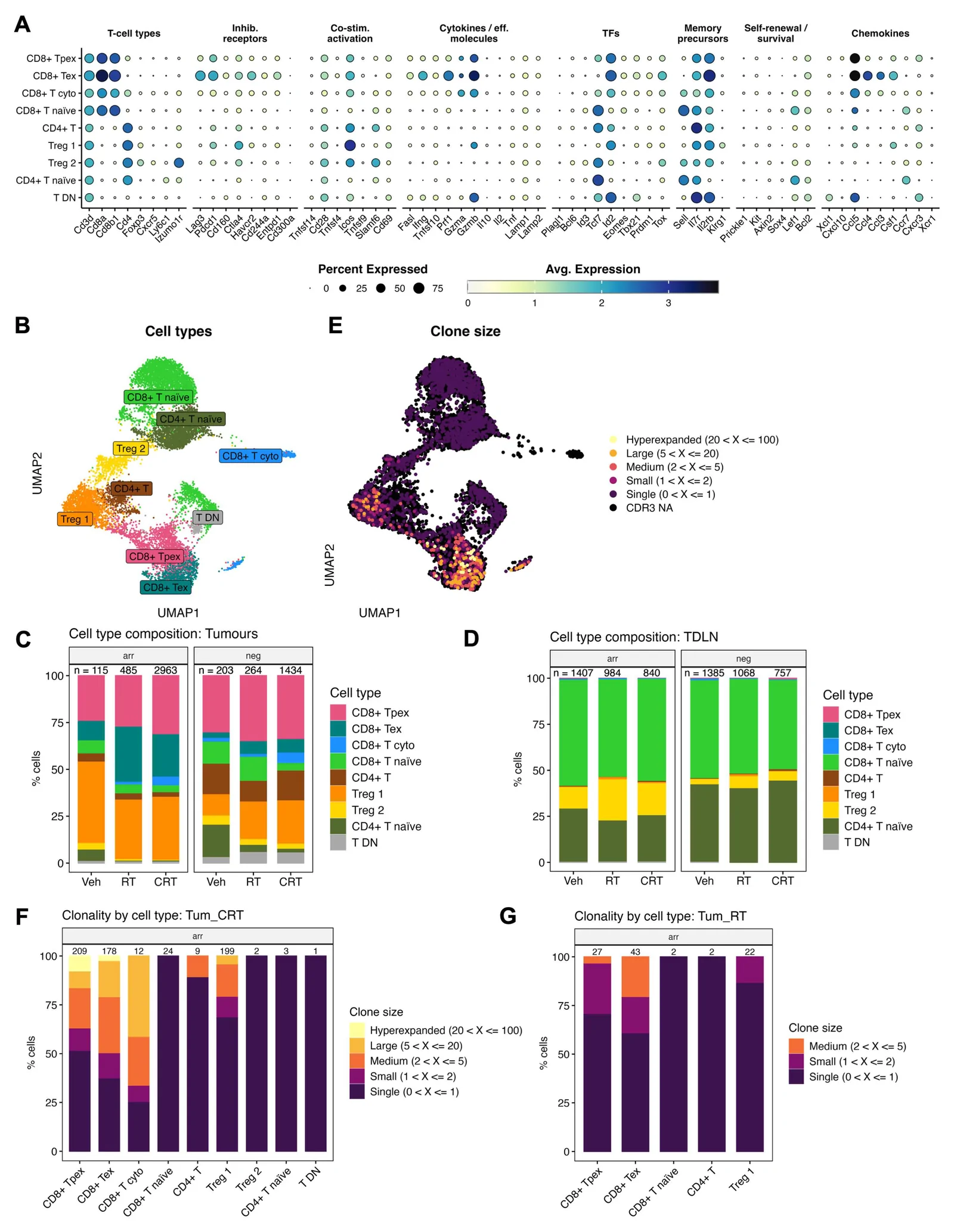
CRT increases the proportion and clonal expansion of precursor exhausted (T_PEX_) to terminally exhausted (T_EX_) CD8+ TILs in the arrested phase, compared to RT alone. Single-cell analysis of samples pooled by treatment group and tissue type, whereby cells were subject to UMAP clustering based on transcriptomic profile (Vehicle n=12, RT n=12, CRT n=12 mice, 1 experiment; only available for Tocky arrested [“arr”] and timer negative [“neg”] samples). **A**. Dot plot illustrating relative gene expression across different T-cell subgroups (each corresponding to a UMAP cluster) and gene categories. Dot colour intensity represents the average expression level, while dot size indicates the percentage of cells expressing the gene. **B**. Cell annotation of clusters by T-cell subgroups. **C-D**. Cell type composition by treatment group and Tocky stage “arr” or “neg” of tumour samples **(C)** and tumour-draining lymph nodes (TDLN) **(D). E**. Clone size categories indicated by clone frequency within the total TCR repertoire: hyperexpanded (>20 and ≤100), large (>5 and ≤20), medium (>2 and ≤5), small (>1 and ≤2) and single (>0 and ≤1). Cells without CDR3 sequence information are denoted by “CDR3 NA”. **F-G:** Barplots illustrating percentage of cells within each clone size category by cell type cluster for “arr” Tum_CRT **(F)** and Tum_RT **(G)**. Abbreviations: Tpex; precursor exhausted T-cells, cyto, cytotoxic; Tex, terminally exhausted T-cells; Treg, regulatory T-cells; T DN, double negative T-cells; Inhib. Receptors, inhibitory receptors; Co-stim. activation; co-stimulatory/ activation; eff. molecules; effector molecules; TFs, transcription factors.

As shown in Fig. 6A-B, naïve CD4^+^ and CD8^+^ T-cells were identified by high expression of CD62L (*Sell*). Classical CD8^+^ T_PEX_ cells were characterized by elevated TCF1 (*Tcf7*), intermediate PD-1 (*Pdcd1*), and low TIM-3 (*Havcr2*) and CD62L (*Sell*), indicative of antigen experience with preserved progenitor features (4, 6, 38). CD8^+^ T_EX_ cells expressed low TCF1, but high TOX, PD-1, LAG-3, and TIM-3, with interestingly maintained Granzyme B (*Gzmb*) and perforin (*Prf1*) expression expression. CD8^+^ cytotoxic (“cyto”) cells exhibited high expression of granzymes (*Gzma, Gzmb*) and perforin (*Prf1*), not falling into the T_PEX_ or T_EX_ phenotype. Two distinct FOXP3-enriched Treg-like subsets were identified: Treg1, displaying an effector/suppressive phenotype (high PD-1, CTLA-4, ICOS, IL2RB and ID2), and Treg2, with a more naïve/central-memory profile (high CD62L and IL7R). Both states correspond to previously described functional Treg phenotypes documented in regulatory T-cell heterogeneity studies (39, 40).

Consistent with AbSeq profiling, TILs were enriched for antigen-experienced populations, including CD8^+^ T_PEX_, CD8^+^ T_EX_, and Treg1 (Fig. 6C), whereas TDLNs were dominated by naïve CD8^+^ T-cells and Treg2 (Fig. 6D). Within the arrested TILs compartment, both RT and CRT increased the frequency of CD8^+^ T_EX_ compared to vehicle, with RT inducing a larger increase. Conversely, CRT preferentially expanded CD8^+^ T_PEX_ compared to RT (Fig. 6C). In the timer-negative compartment, both RT and CRT similarly enhanced the proportion of T_PEX and_ T_EX_ compared to vehicle. These findings suggest that, in cells with a history of antigen engagement, CRT skews differentiation toward progenitor-like states, while RT promotes more terminal T-cell exhaustion.

To explore transcriptional clonal dynamics, we integrated paired TCR VDJ and scRNA-seq profiles, mapping clonotypes onto UMAP-defined subsets (Fig. 6E). Clonal expansion occurred mainly in “arrested” TILs rather than T-cells from TDLNs (Fig. S14). As shown in Fig. 6F, large and hyperexpanded clones were exclusively observed in CRT-treated TILs, predominantly within CD8^+^ T_PEX_ and, to a lesser extent, CD8^+^ T_EX_ and Treg1 subsets. Notably, the majority of hyperexpanded clones resided in the T_PEX_ population, whereas RT-induced expansion was restricted to smaller clones within T_EX_ clusters (Fig. 6G). Thus, CRT promotes robust clonal expansion of T_PEX_ cells, while RT favours expansion of terminally exhausted subsets.

Overall, compared to vehicle, CRT reduced the proportion of T_EX_ CD8^+^ TILs and increased T_PEX_ populations, whereas RT drove the opposite trend. These results, supported by lineage and clonotype mapping, indicate that CRT preferentially maintains and/or expands progenitor-exhausted states, potentially preserving antitumour functionality compared to RT alone.

### Enhanced T-cell activation and altered exhaustion dynamics after CRT versus RT in human PBMCs

**Fig. 7:**
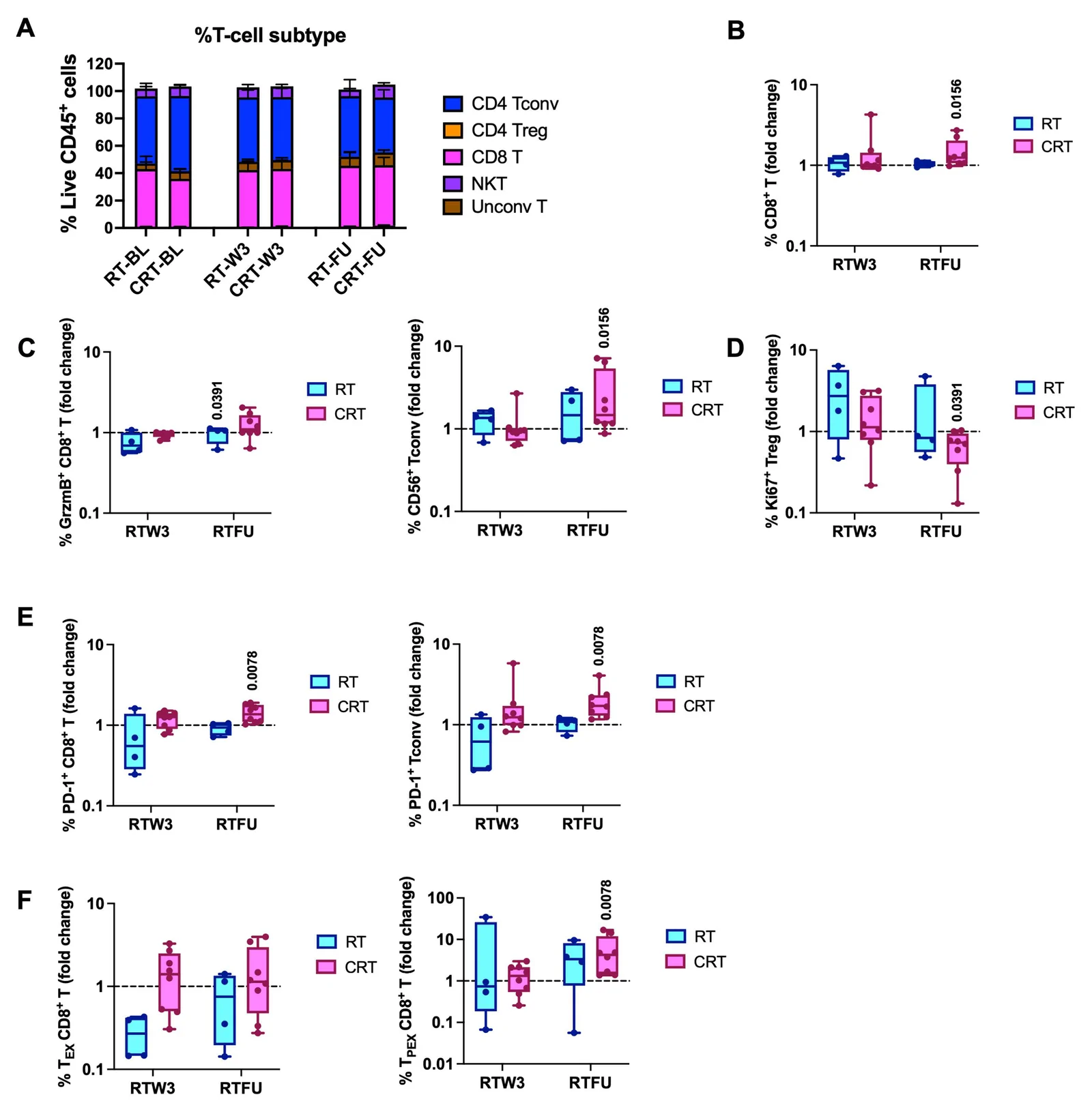
CRT versus RT differentially modulates T-cell subsets and exhaustion trajectories in human PBMCs. Peripheral blood mononuclear cells (PBMCs) were collected from patients with LA-SCCHN receiving definitive RT (n=4) or CRT (n=8) as part of the INOVATE study (ISRCTN32335415) at baseline (BL), week 3 of RT (RTW3), and 3 months post-treatment (RTFU; one RT patient at 6 weeks). Flow cytometry analysis of PBMCs was performed. **A**. Frequencies of T-cell subsets over time, comparing RT and CRT. **B-F**. Fold changes relative to baseline at RTW3 and RTFU for CD8^+^ T-cells **(B)**, Granzyme B^+^ (GrzmB^+^) CD8^+^ T-cells and CD56^+^ Tconv **(C)**, Ki67^+^ Tregs **(D)**, PD-1^+^ CD8^+^ and Tconv **(E)**, terminally exhausted (T_EX_; TOX^+^ TCF-1^−^ PD-1^hi^) and progenitor exhausted (T_PEX_; TCF-1^+^ TOX^−^ PD-1^int^) CD8^+^ T-cell populations **(F)**. Box-and-whisker plots show the median, interquartile range (25^th^-75^th^ percentiles), and minimum-to-maximum whiskers, with individual data points shown. Statistical significance for fold change comparisons in panels B-F was assessed using the Wilcoxon signed-rank test; *p*<0.05 was considered statistically significant.

To provide proof-of-concept translational validation of CRT’s impact on the T-cell exhaustion trajectory versus RT, we analysed a small exploratory cohort of HPV-positive patients with LA-SCCHN receiving definitive RT (n=4) or CRT (n=8) as part of the INOVATE study (ISRCTN32335415). Patient clinical characteristics are summarised in Table 1. Peripheral blood mononuclear cells (PBMCs) were collected from patients receiving RT or CRT at baseline, week 3 of RT (RTW3), and 3 months post-treatment (RTFU), and analysed by flow cytometry. Over time, there was a trend toward a reduction in CD4^+^ Tconv and an increase in CD8^+^ T-cell frequencies related to CD45^+^ population, more pronounced in CRT than RT (Fig. 7A). At the 3-month follow-up, only CRT induced a significant fold change in CD8^+^ T-cells relative to baseline (Fig. 7B). CRT was also associated with increased Granzyme B^+^ CD8^+^ T-cells and CD56^+^ NK-like CD4^+^ Tconv (41–43) at follow-up, indicative of enhanced cytotoxic activity (Fig. 7C). In parallel, proliferating Ki67^+^ Treg were significantly reduced after CRT but not RT (Fig. 7D), while PD-1 expression on both CD4^+^ Tconv and CD8^+^ T-cells was increased after CRT (Fig. 7E). Examination of exhaustion marker trajectories revealed that both RT and CRT showed trends toward increasing putative T_EX_ CD8^+^ T-cells (TOX^+^ TCF-1^−^ PD-1^hi^) (44–46) during treatment (Fig. 7F). Notably, a significant increase in putative T_PEX_ cells (TCF-1^+^ TOX^−^ PD-1^int^) (5, 46, 47) emerged only after CRT at the 3-month follow-up (Fig. 7F). Hence, even with this limited number of patients, these data revealed longitudinal differences between RT and CRT in peripheral T-cell subset composition, cytotoxic activity, and exhaustion trajectories that mirror the data generated in the mouse model. The distinct CD8^+^ T-cell responses observed following RT and CRT across the preclinical and human analyses are summarised schematically in Fig. S15.

## Discussion

This study demonstrates that CRT and RT induce distinct intratumoural T-cell responses in a relevant SCCHN tumour model. Functional depletion experiments establish that tumour control under CRT, but not RT, is CD8^+^ T-cell dependent, confirming a central role for adaptive immunity in CRT efficacy. Consistent with this, mice achieving complete remission following CRT exhibited enhanced protection against tumour rechallenge, supporting the generation of durable systemic antitumour immunity. Using the Nr4a3-Tocky reporter system integrated with single-cell transcriptomic, protein, and TCR clonotype profiling, we show that CRT enhances antigen engagement, promotes robust CD8^+^ T-cell clonal expansion, and biases differentiation toward a progenitor exhausted (T_PEX_) state rather than terminal exhaustion (T_EX_).

CRT induced a qualitatively distinct T-cell response compared with RT. Although both treatments increased immune activation and checkpoint expression, CRT generated a more focused and persistent CD8^+^ T-cell repertoire, including hyperexpanded clonotypes restricted to a small number of dominant TCR α/β pairs. Integration of TCR and transcriptomic data demonstrated that large and hyperexpanded clones were predominantly observed following CRT, whereas RT-associated clonal expansion was largely restricted to smaller clones. These findings indicate that CRT promotes more extensive antigen-driven clonal expansion than RT.

Single-cell transcriptomics confirmed distinct T_EX_ and T_PEX_ states across tumour-infiltrating lymphocytes (TILs) and revealed treatment-specific patterns. CRT preferentially enriched T_PEX_ cells characterised by high *Tcf7*, intermediate *Pdcd1*, and reduced exhaustion markers, while RT promoted accumulation of terminally exhausted states (T_EX_). Integration of transcriptomic and TCR data demonstrated that large and hyperexpanded clones were largely confined to CRT-treated tumours and preferentially localised within T_PEX_ clusters. In contrast, RT-associated clonal expansion was predominantly localised within T_EX_ populations. Together, these findings indicate that CRT promotes clonal expansion within T_PEX_ populations, whereas RT favours expansion of T_EX_ subsets.

A notable finding was the emergence of a clonally expanded Treg-cell-like population under CRT with a suppressive transcriptional profile, including elevated PD-1, CTLA-4, ICOS, IL2RB, and ID2 expression. This may partially counterbalance effector T-cell activity and provide a mechanistic explanation for the limited efficacy of PD-1 blockade observed in this setting in both our preclinical model and prior studies. These data suggest that additional targeting of immunosuppressive compartments may be necessary to maximise the efficacy of immunotherapy in combination with CRT.

Interestingly, no shared clonotypes were detected between TDLNs and tumours, possibly due to limited sequencing depth, pooled sampling across animals, or the timing of tissue collection. However, shared clonotypes along the Tocky trajectory within TILs support antigen engagement as a driving force for clonal expansion within tumours.

Translationally, these findings suggest that CRT reshapes systemic and local immunity beyond direct cytotoxicity. In human PBMCs, CRT was associated with sustained expansion of terminally exhausted CD8^+^ T-cells during treatment, followed by delayed emergence of T_PEX_-like populations at later time points. This temporal separation may reflect a post-treatment recovery phase that could be critical for subsequent immune reinvigoration. CRT was also associated with increased cytotoxic potential and reduced proliferating Tregs, supporting a shift toward a more activated systemic immune state. However, as these analyses were restricted to peripheral blood, they do not fully capture the intratumoural dynamics observed in preclinical models, and spatial compartmentalisation remains an important limitation. Clinically, these findings align with emerging evidence that concurrent immune checkpoint inhibition (ICI) with CRT has limited efficacy in unresected LA-SCCHN (18–20). One explanation may be the early dominance of exhausted T-cell states in the periphery, which may constrain effective reinvigoration of newly emerging progenitor populations. These observations raise the possibility that temporal changes in T-cell state following CRT may influence responses to subsequent immunotherapy and warrant further investigation in prospective studies.

Limitations of this study include limited single-cell RNA sequencing depth which constrained detection of low-abundance transcripts and fine-grained trajectory inference. Technical limitations of the multiomic workflow resulted in insufficient recovery of cells from the “new” and “persistent” Nr4a3-Tocky populations for scRNA-seq analysis. Consequently, transcriptional comparison of T_PEX_ and T_EX_ T-cell states across the full Tocky trajectory was not possible. The use of pooled tumour and TDLN samples may have masked inter-individual clonal relationships, and the exclusive focus on CD45^+^/T-cell-enriched populations limits assessment of broader tumour microenvironmental interactions. In the exploratory clinical cohort, sample size was modest, and analyses were restricted to peripheral blood rather than tumour tissue. In addition, the limited availability of longitudinal PBMC samples did not permit direct assessment of T-cell receptor signalling and activation. Furthermore, although our murine chemoradiotherapy model enabled controlled mechanistic dissection of treatment-induced immune responses, it does not fully recapitulate definitive clinical chemoradiotherapy. The hypofractionated radiation regimen (8 Gy × 3), single-dose cisplatin schedule and subcutaneous tumour model differ from the conventionally fractionated chemoradiotherapy delivered to patients with head and neck cancer affecting the upper aerodigestive tract. Accordingly, while the accompanying longitudinal PBMC analyses provide translational support for treatment-associated immune changes in patients, the mechanistic insights derived from the murine model should be interpreted within the context of this experimental treatment paradigm. Future longitudinal studies incorporating orthotopic models, conventionally fractionated CRT, paired tumour-TDLN sampling, assessment of TCR signalling in patient PBMCs, and deeper multiomic profiling with improved recovery of all Nr4a3-Tocky populations will be required to resolve how temporal TCR signalling relates to T-cell differentiation states and to fully define the spatial and temporal immune dynamics following CRT.

Future studies should investigate whether treatment-induced changes in T-cell state can be leveraged to optimise immunotherapy sequencing and patient selection. Rational combinatorial treatment strategies that move beyond ICI alone with CRT, could include Treg depletion (e.g. anti-CD25) (48), targeting immunosuppressive myeloid populations (49), or radiosensitising agents that enhance immunogenic cell death to further amplify antigen release and CD8^+^ T-cell priming (50). Such combinations may help overcome residual immunosuppressive barriers and improve the durability of responses to CRT combined with ICI.

In conclusion, CRT reshapes the tumour immune landscape by promoting antigen-engaged CD8^+^ T-cell expansion, sustaining T_PEX_ states, and restricting T_EX_ differentiation. These data provide insight into how CRT modulates CD8^+^ T-cell fate and clonal architecture and reveal distinct immune trajectories induced by CRT and RT in SCCHN.

## Supplementary Material

1

2

3

4

5

6

7

8

9

10

11

12

13

14

15

16

## Figures and Tables

**Table 1:** Clinical characteristics of patients included in the longitudinal PBMC analysis.

	CRT (n=8)	RT (n = 4)
**Patient demographics**		
**Age, years, median (range)**	58 (50-74)	68 (52-75)
**Sex**		
**Male (%)**	8 (100%)	3 (75%)
**Female (%)**	0 (0%)	1 (25%)
Tumour (T) stage* **(%)**		
**T1**	1 (12.5%)	1 (25%)
**T2**	3 (37.5%)	1 (25%)
**T3**	1 (12.5%)	1 (25%)
**T4**	3 (37.5%)	1 (25%)
Nodal (N) stage* **(%)**		
**N1**	7 (87.5%)	4 (100%)
**N2**	1 (12.5%)	0 (0%)
Stage group* **(%)**		
**I**	4 (50%)	2 (50%)
**II**	1 (12.5%)	1 (25%)
**III**	3 (37.5%)	1 (25%)

* Stage according to the AJCC TNM Staging Manual, 8th edition, for HPV-positive (p16-positive) oropharyngeal squamous cell carcinoma. All patients had M0 disease.

## Data Availability

The multiome sequencing data (joint single-cell RNA, TCR, and AbSeq profiling) generated in this study using the BD Rhapsody platform have been deposited in the NCBI Gene Expression Omnibus (GEO; RRID:SCR_005012) under accession number GSE329670.

## Synopsis

Here, the authors report that concomitant chemoradiotherapy (CRT) impacts antitumor T-cell responses in head and neck cancer, with CRT promoting antigen-engaged CD8^+^ T-cell expansion while sustaining the T_PEX_ state and restricting T_EX_ differentiation.
