# Supplementary material for: Addition of chemotherapy to radiotherapy promotes progenitor-exhausted CD8+ T-cell clonal dominance in head and neck cancer
Source: Cancer Immunol Res. Author manuscript; Available in PMC 2026 Nov 5. (PMC7619705; doi:10.1158/2326-6066.CIR-26-0706)
Supplement: Supplementary file 1 [file EMS220945-supplement-1.pdf]

Figure S1

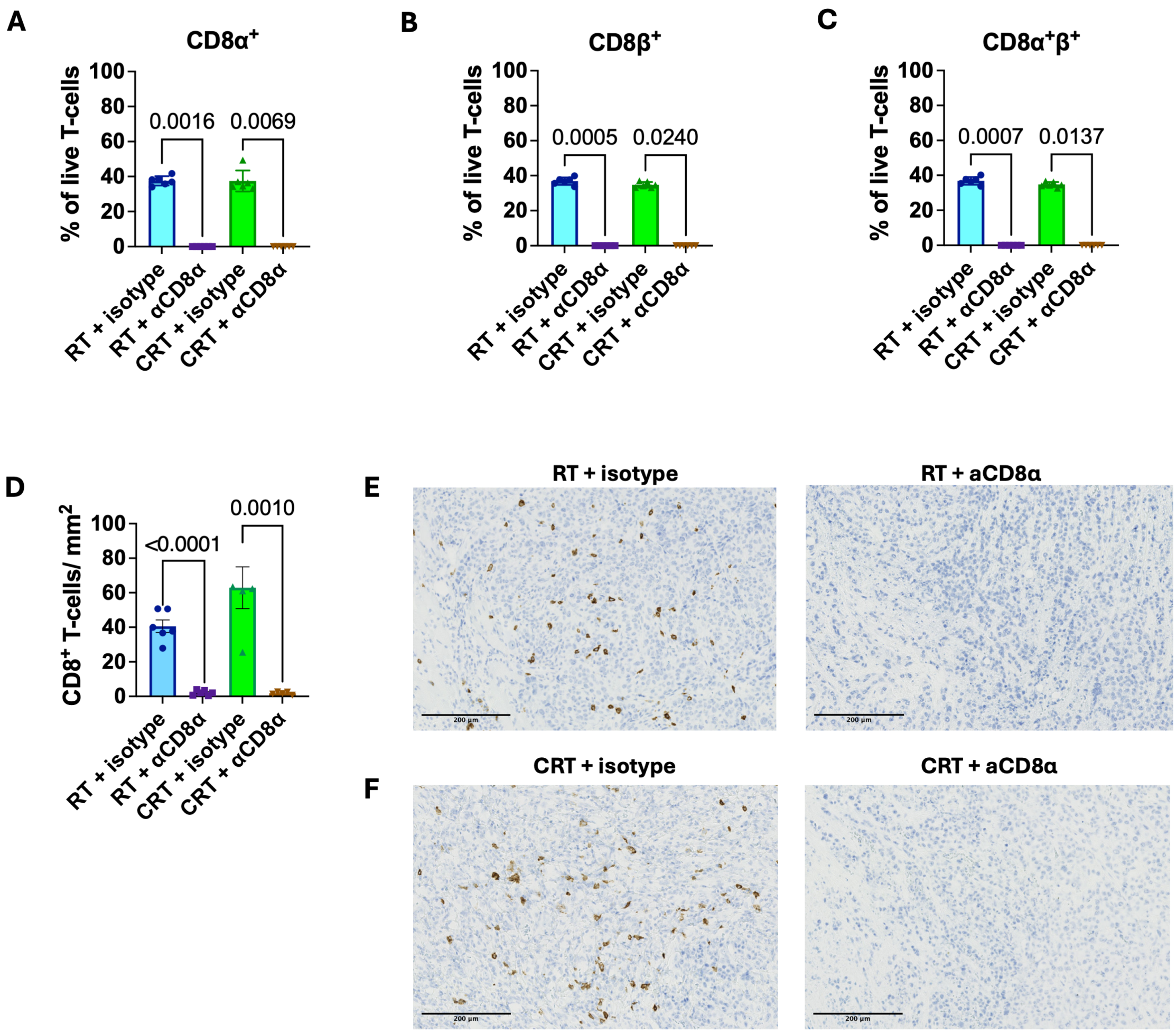

Fig. S1: Confirmation of peripheral blood and intratumoural $CD8^+$ T-depletion *in vivo* administration of a depleting anti-CD8α antibody.
A-C: Peripheral blood from the tail vein of all mice from indicated treatment groups was collected two weeks following initiation of *in vivo* CD8α depletion. Flow cytometry was performed to assess presence of **(A)** $CD8\alpha^+$ **(B)** $CD8\beta^+$ and **(C)** $CD8\alpha^+\beta^+$ T-cells. **D-F:** Tumours were collected at day 50 after tumour cell implantation and αCD8α immunohistochemistry (IHC) was performed. **(D)** Quantification of $CD8^+$ T-cells per mm^2 tumour area in αCD8α-treated versus isotype control-treated tumours. Representative IHC images were taken from **(E)** RT + isotype/αCD8α, **(F)** CRT + isotype/αCD8α. Sample sizes: RT + isotype n=6, RT + αCD8 n=6. CRT + isotype n=6, CRT + αCD8α n=6; panel D: RT + isotype n=6, RT + αCD8 n=6. CRT + isotype n=5, CRT + αCD8α n=5. Data are from one experiment.
