# Supplementary material for: Addition of chemotherapy to radiotherapy promotes progenitor-exhausted CD8+ T-cell clonal dominance in head and neck cancer
Source: Cancer Immunol Res. Author manuscript; Available in PMC 2026 Nov 5. (PMC7619705; doi:10.1158/2326-6066.CIR-26-0706)
Supplement: Supplementary file 2 [file EMS220945-supplement-10.pdf]

Figure S10

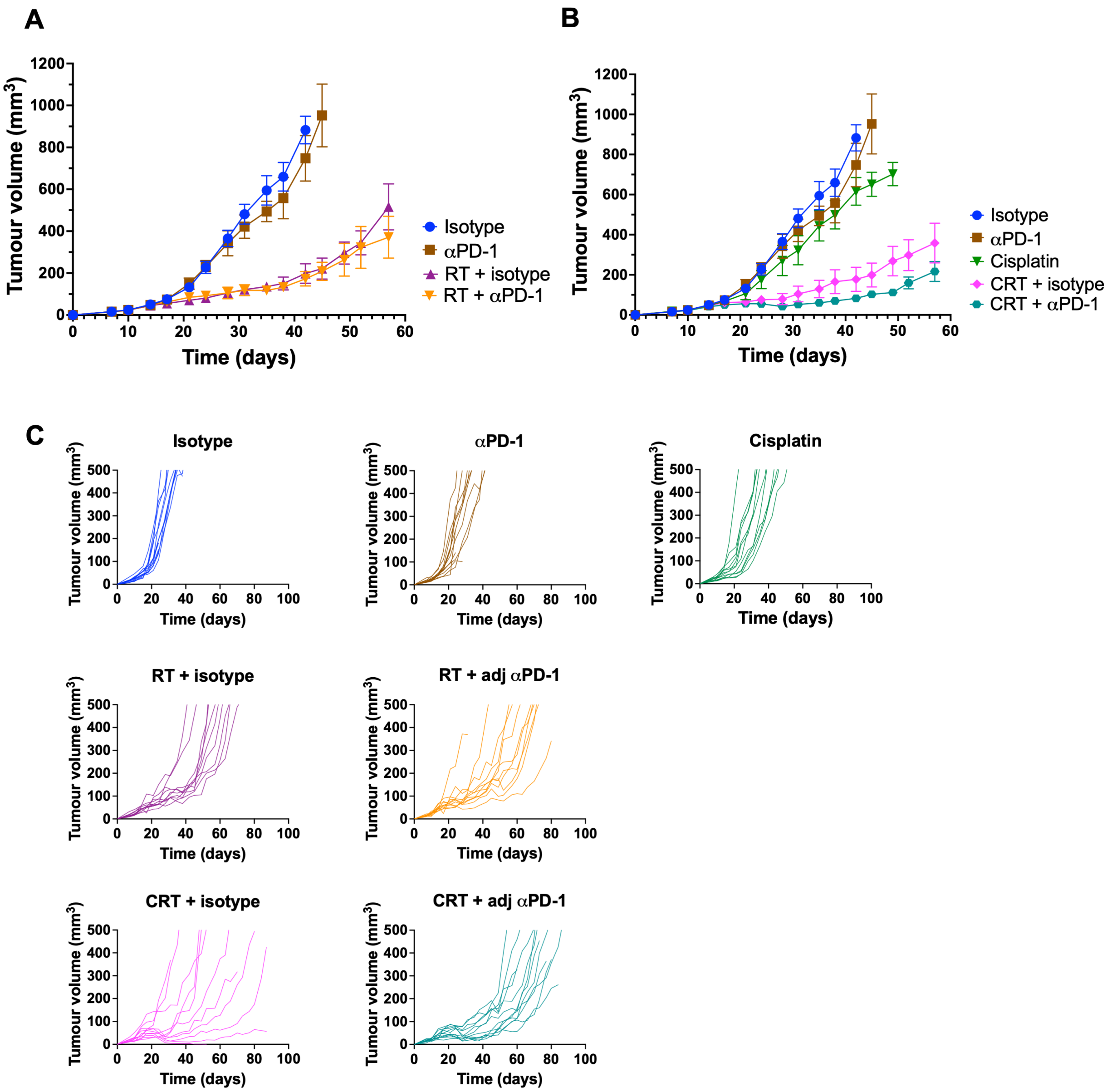

Fig. S10: *In vivo* effect of anti-PD-1 antibody on RT and CRT in the mEER model.
A-B: Tumour growth curves across indicated treatment conditions: **(A)** RT plus anti-PD-1 experiment, (**B)** CRT plus anti-PD-1. **C.** Individual tumour growth curves (Isotype n=12, αPD-1 n=12, RT + isotype n=10, Cisplatin n=12, CRT + isotype n=11, RT + αPD-1 n=11, CRT + αPD-1 n=12; 2 independent experiments).
