# Supplementary material for: Addition of chemotherapy to radiotherapy promotes progenitor-exhausted CD8+ T-cell clonal dominance in head and neck cancer
Source: Cancer Immunol Res. Author manuscript; Available in PMC 2026 Nov 5. (PMC7619705; doi:10.1158/2326-6066.CIR-26-0706)
Supplement: Supplementary file 3 [file EMS220945-supplement-11.pdf]

Figure S11

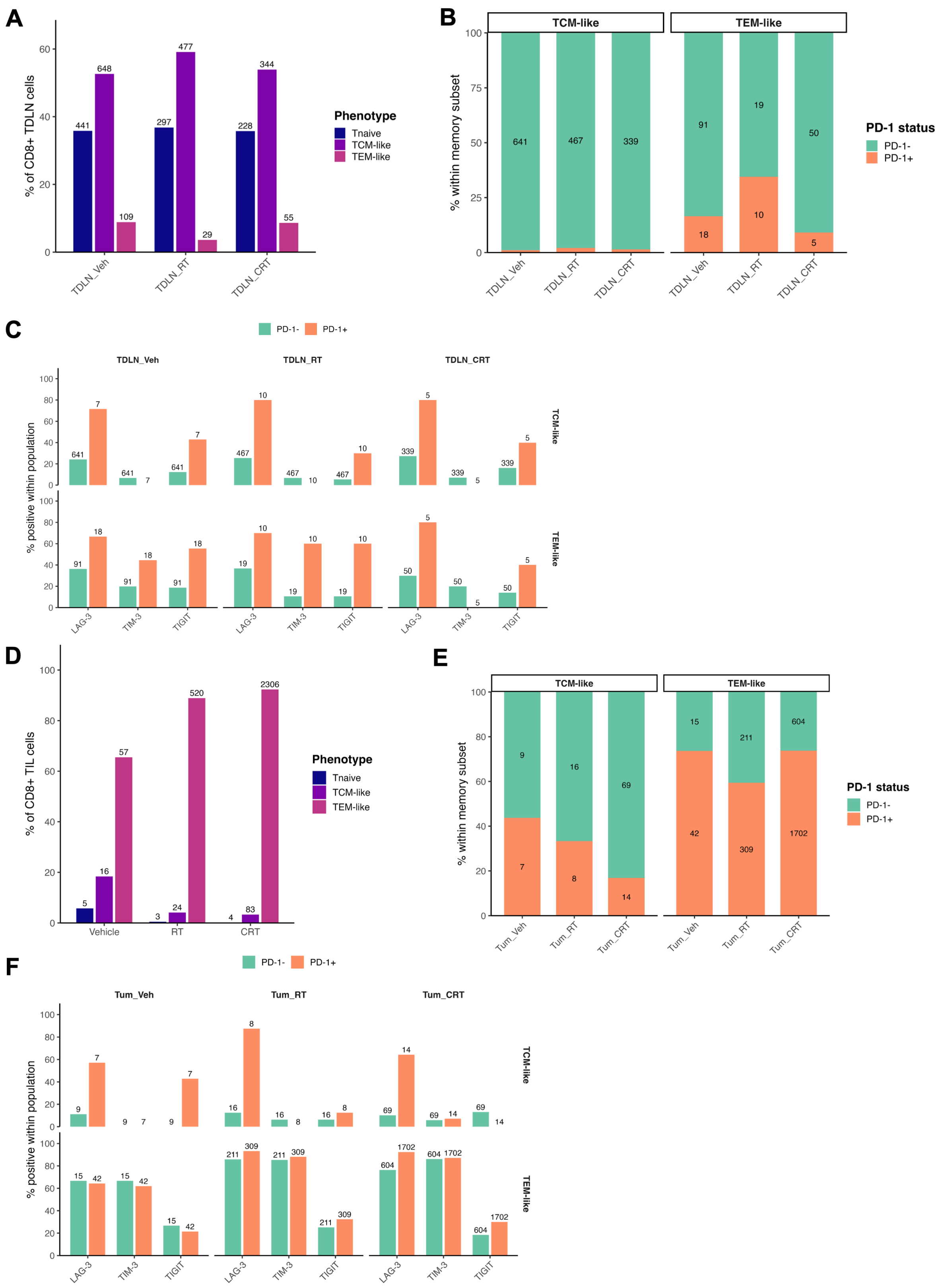

Fig. S11. PD-1 stratification of memory-like CD8⁺ T-cell populations in TDLNs and TILs. Single-cell AbSeq analysis was performed on sorted CD8$^+$ T cells from tumours and TDLNs harvested from mEER-bearing Nr4a3-Tocky mice treated with vehicle (Veh), RT or CRT (Vehicle n = 12, RT n = 12, CRT n = 12; 1 experiment). **A-C.** TDLN analyses showing **(A)** the proportion of TN-, TCM- and TEM-like CD8$^+$ T-cell populations across treatment groups; **(B)** the relative proportions of PD-1$^+$ and PD-1$^-$ cells within TCM- and TEM-like populations; and **(C)** the percentage of PD-1$^+$ and PD-1$^-$ TCM- and TEM-like cells expressing LAG-3, TIM-3 or TIGIT. **D-F.** Corresponding analyses of TILs.
