# Supplementary material for: Addition of chemotherapy to radiotherapy promotes progenitor-exhausted CD8+ T-cell clonal dominance in head and neck cancer
Source: Cancer Immunol Res. Author manuscript; Available in PMC 2026 Nov 5. (PMC7619705; doi:10.1158/2326-6066.CIR-26-0706)
Supplement: Supplementary file 4 [file EMS220945-supplement-12.pdf]

Figure S12

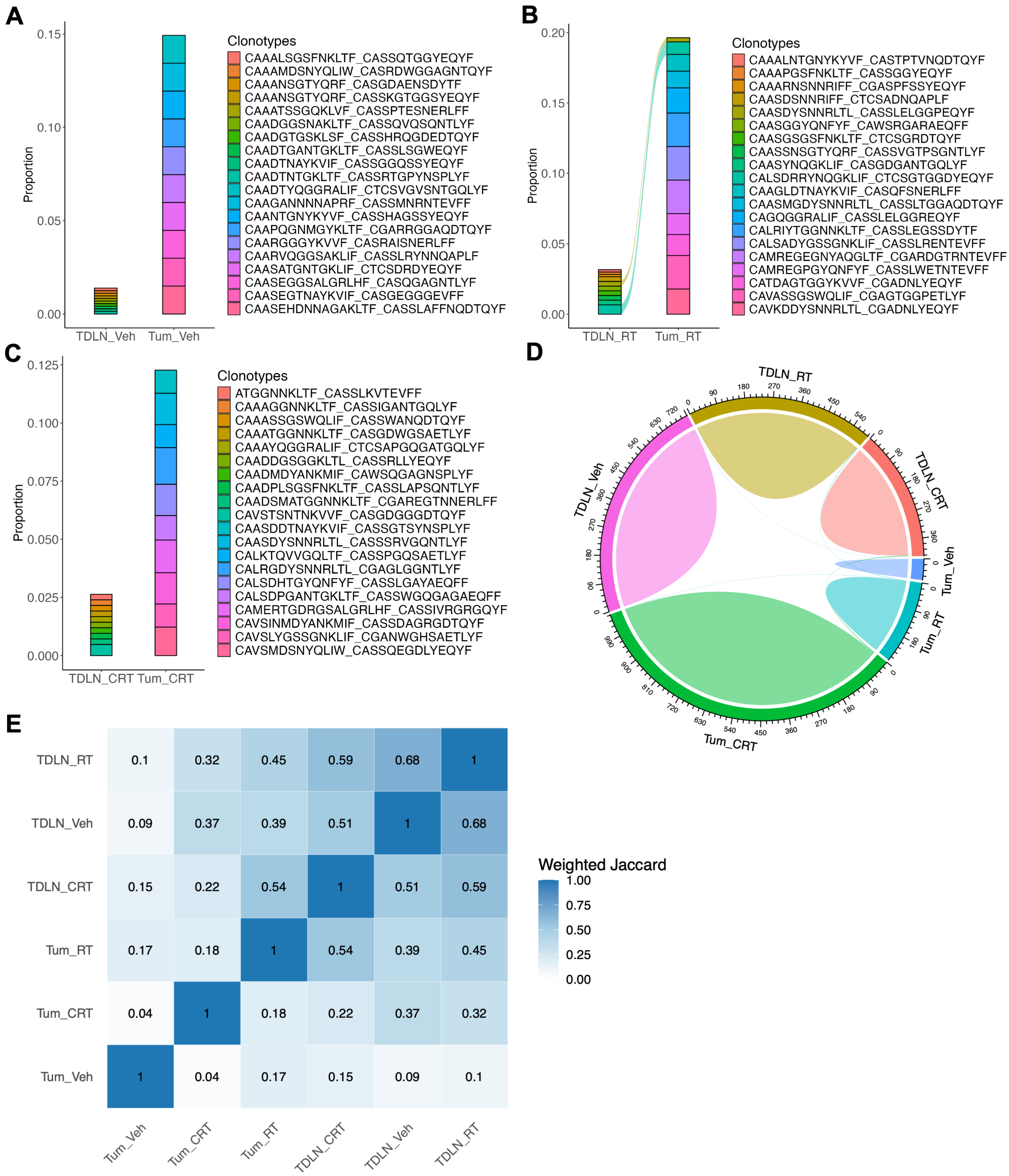

Fig. S12: Lack of shared clonotypes between tumour and TDLN sample types of the same treatment group.
Single-cell TCR-seq analysis was performed on sorted tumour-infiltrating lymphocytes (TILs) from tumours (Tum) or TDLN harvested from mEER-bearing mice treated with vehicle (Veh), RT or CRT (Vehicle n=12, RT n=12, CRT n=12, 1 experiment). **A-C.** Alluvial plots tracking the top 10 clonotypes between sample types (Tumour [Tum] and TDLN) of the same treatment group: Vehicle **(A)**, RT **(B)**, CRT **(C)**. **D.** Chord diagram illustrating lack of shared clonotypes between all samples. **E.** Frequency-weighted 3-mer Jaccard similarity heatmap, visualising the degree of TCR repertoire similarity between samples.
