# Supplementary material for: Addition of chemotherapy to radiotherapy promotes progenitor-exhausted CD8+ T-cell clonal dominance in head and neck cancer
Source: Cancer Immunol Res. Author manuscript; Available in PMC 2026 Nov 5. (PMC7619705; doi:10.1158/2326-6066.CIR-26-0706)
Supplement: Supplementary file 5 [file EMS220945-supplement-13.pdf]

Figure S13

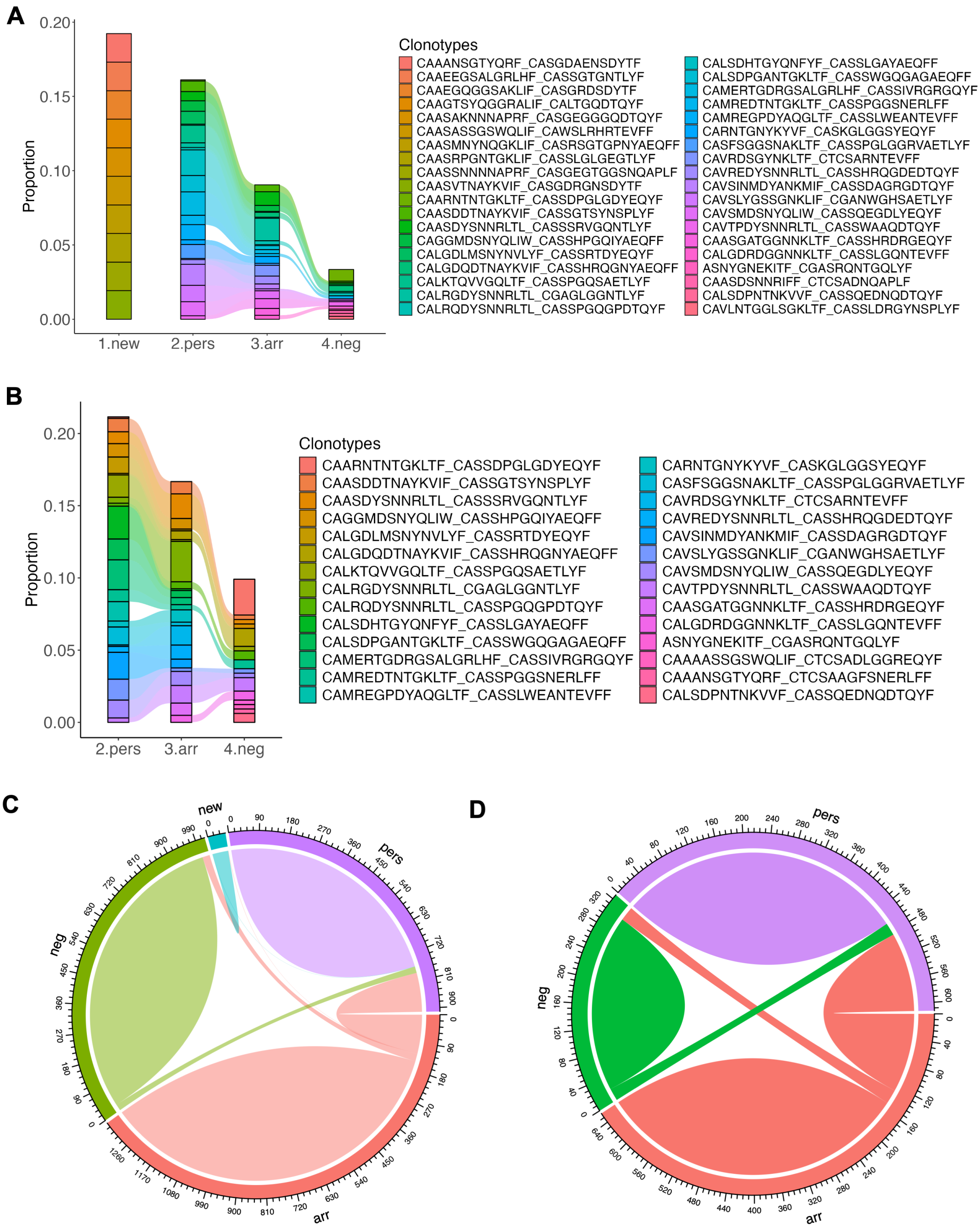

Fig. S13: Shared clonotypes are seen to persist according to the Tocky trajectory of antigen engagement.

Single-cell TCR-seq analysis was performed on sorted tumour-infiltrating lymphocytes (TILs) from tumours (Tum) or TDLN harvested from mEER-bearing mice treated with vehicle (Veh), RT or CRT (Vehicle n=12, RT n=12, CRT n=12, 1 experiment). **A-B.** Alluvial plots tracking the top 10 clonotypes across the different Tocky populations: “new”, “persistent” (pers), “arrested” (arr) and “timer negative” (neg), for all samples (Tumour and TDLN) **(A)** and Tumour samples alone **(B). C-D.** Chord diagrams showing shared clonotypes across different Tocky populations, for all samples (Tumour and TDLN) **(C)** and Tumour samples alone **(D).**
