# Supplementary material for: Addition of chemotherapy to radiotherapy promotes progenitor-exhausted CD8+ T-cell clonal dominance in head and neck cancer
Source: Cancer Immunol Res. Author manuscript; Available in PMC 2026 Nov 5. (PMC7619705; doi:10.1158/2326-6066.CIR-26-0706)
Supplement: Supplementary file 6 [file EMS220945-supplement-14.pdf]

Figure S14

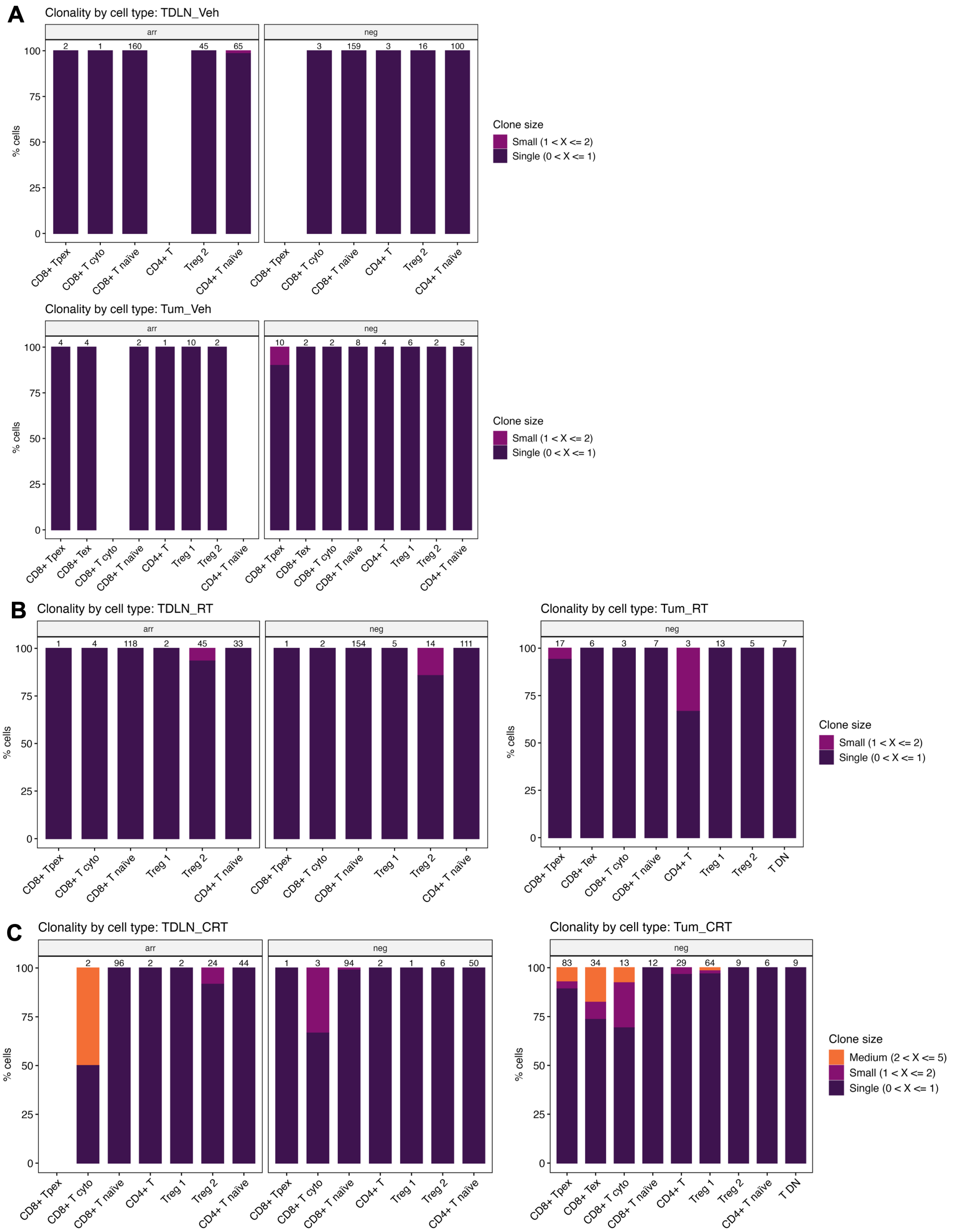

Fig. S14: Clonality group by sample type, cell type and Tocky loci.
Single-cell analysis whereby cells were subject to UMAP clustering based on transcriptomic profile (Vehicle n=12, RT n=12, CRT n=12, 1 experiment; available for Tocky arrested [“arr”] and timer negative [“neg”] samples). **A-C:** Barplots illustrating percentage of cells within each clone size category by cell type cluster for **(A)** Vehicle-treated TDLN (TDLN_Veh) and tumours (Tum_Veh), **(B)** RT-treated TDLN (TDLN_RT) and tumours (Tum_RT) [Tum_RT “arr” data displayed in Fig. 6G] and **(C)** CRT-treated TDLN (TDLN_CRT) and tumours (Tum_CRT) [Tum_CRT ”arr” data displayed in Fig. 6F]. Abbreviations: Tpex; precursor exhausted T-cells, cyto, cytotoxic; Tex, terminally exhausted T-cells; Treg, regulatory T-cells; T DN, double negative T-cells; Inhib. Receptors, inhibitory receptors; Co-stim. activation; co-stimulatory/ activation; eff. molecules; effector molecules; TFs, transcription factors; cyto, cytotoxic; Treg, regulatory T-cells.
