# Supplementary material for: Addition of chemotherapy to radiotherapy promotes progenitor-exhausted CD8+ T-cell clonal dominance in head and neck cancer
Source: Cancer Immunol Res. Author manuscript; Available in PMC 2026 Nov 5. (PMC7619705; doi:10.1158/2326-6066.CIR-26-0706)
Supplement: Supplementary file 7 [file EMS220945-supplement-15.pdf]

Figure S15

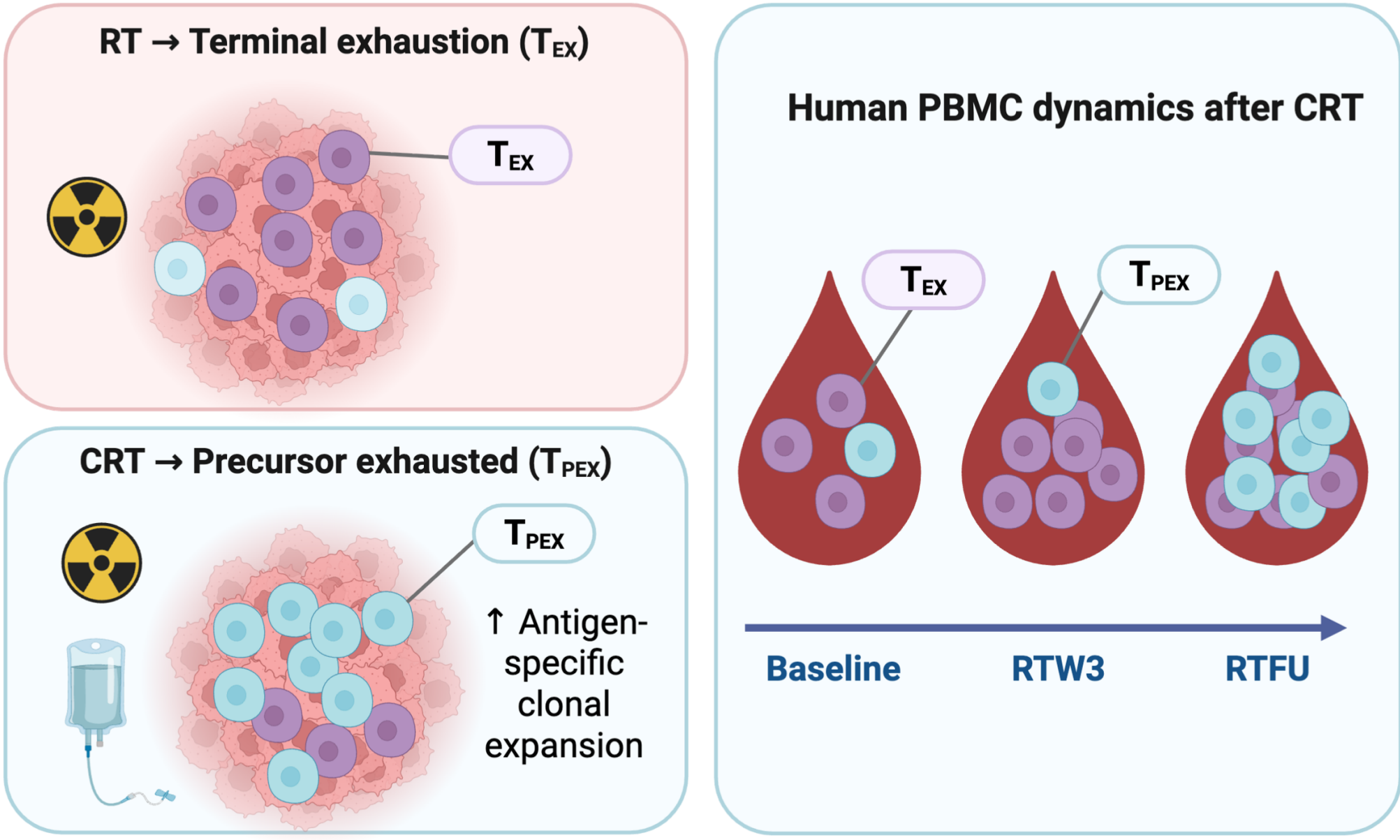

Fig. S15: Distinct CD8+ T-cell remodelling by chemoradiotherapy versus radiotherapy. Graphical abstract illustrating how CRT, compared to RT alone, differentially shapes CD8+ T- cell states in pre-clinical models of head and neck squamous cell carcinoma. CRT drives progenitor-exhausted (T_{PEX}) clonal expansion within tumours, while RT favours terminal exhaustion (T_{EX}). Human PBMC analyses following CRT show delayed recovery of T_{PEX} cells 3 months post-RT (RTFU). RTW3: during week 3 of RT. *Created in BioRender. Harrington, K. (2026) https://BioRender.com/e3va31y.*
