# Supplementary material for: Addition of chemotherapy to radiotherapy promotes progenitor-exhausted CD8+ T-cell clonal dominance in head and neck cancer
Source: Cancer Immunol Res. Author manuscript; Available in PMC 2026 Nov 5. (PMC7619705; doi:10.1158/2326-6066.CIR-26-0706)
Supplement: Supplementary file 8 [file EMS220945-supplement-16.pdf]

Table S1. List of anti-mouse antibodies used for immunoprofiling

Epitope	Clone	Fluorophore	Manufact-urer	Catalog number	RRID	Dilution
**CD103**	M290	BUV805	BD	741948	RRID:AB_2871259	1:100
**CD11b**	M1/70	BV750	Biolegend	101267	RRID:AB_2810328	1:200
**CD11c**	N418	BUV615	BD	751222	RRID:AB_2875243	1:100
**CD19**	ID3	BUV395	BD	565965	RRID:AB_2739418	1:200
**CD25**	PC61	AF700	Biolegend	102024	RRID:AB_493709	1:100
**CD3**	17A2	BUV563	BD	741319	RRID:AB_2870837	1:100
**CD4**	RM4-5	APC	Biolegend	100515	RRID:AB_312718	1:200
**CD4**	RM4-4	BUV496	BD	741051	RRID:AB_2870666	1:200
**CD4**	RM4-5	PerCP	Biolegend	100538	RRID:AB_893325	1:100
**CD44**	IM7	BUV395	BD	568507	RRID:AB_3677624	1:100
**CD45**	30-F11	AF700	Biolegend	103128	RRID:AB_493715	1:300
**CD45**	30-F11	BUV805	BD	568336	RRID:AB_3684191	1:300
**CD62L**	MEL-14	BV510	Biolegend	104441	RRID:AB_2561537	1:100
**CD69**	H1.2F3	PE-Cy7	Biolegend	104512	RRID:AB_493564	1:100
**CD8α**	53-6.7	BV785	Biolegend	100750	RRID:AB_2562610	1:100
**CD8α**	53-6.7	PE-Cy7	Biolegend	100721	RRID:AB_312760	1:300
**CD8β**	53-5.8	FITC	Biolegend	140403	RRID:AB_10641694	1:200
**CD80**	16-10A1	BV510	Biolegend	104741	RRID:AB_2810337	1:100
**CD86**	GL-1	APC	Biolegend	105011	RRID:AB_493343	1:100
**EpCAM**	G8.8	AF488	Biolegend	118210	RRID:AB_1134099	1:200
**F4/80**	BM8	PE/Dazzle	Biolegend	123145	RRID:AB_2564132	1:100
**FOXP3**	FJK-16s	eFluor 450	eBioscience	48-5773-82	RRID:AB_1518812	1:100
**Granzyme B**	GB11	AF647	Biolegend	515405	RRID:AB_2294995	1:100
**Ly6C**	HK1.4	AF700	Biolegend	128023	RRID:AB_10640119	1:100
**Ly6G**	1A8	PerCP/Cy5.5	Biolegend	127615	RRID:AB_1877272	1:100
**MHC-II**	M5/114.15.2	BUV496	BD	750281	RRID:AB_2874472	1:200
**NK1.1**	PK136	BUV737	BD	741715	RRID:AB_2871088	1:100
**PD-1**	RMP1-30	APC	Biolegend	109112	RRID:AB_10612938	1:100
**PD-1**	29F.1A12	BV605	Biolegend	135219	RRID:AB_11125371	1:200
**Perforin**	S16009B	PE	Biolegend	154405	RRID:AB_2721640	1:100
**T-bet**	4B10	PE-Cy7	eBioscience	25-5825-82	RRID:AB_11042699	1:100
**TCRβ**	29A1.4	BUV737	BD	612821	RRID:AB_2870145	1:300
**TIM-3**	RMT3-23	BV711	Biolegend	119727	RRID:AB_2716208	1:100
**XCR1**	ZET	BV650	Biolegend	148220	RRID:AB_2566410	1:200

Table S2. List of anti-human antibodies used for immunoprofiling

Epitope	Clone	Fluorophore	Manufact-urer	Catalog number	RRID	Dilut-ion
**CCR7**	G043H7	BUV395	eBioscience	363-1979-41	RRID:AB_2937087	1:17
**CD25**	BC96	RB744	BD	570482	RRID:AB_3685775	1:17
**CD27**	O323	BUV805	eBioscience	368-0279-41	RRID:AB_2896105	1:17
**CD3**	HIT3a	AF700	Biolegend	300324	RRID:AB_493739	1:100
**CD4**	SK3	BV510	Biolegend	344634	RRID:AB_2566017	1:200
**CD45**	HI30	BV650	Biolegend	304044	RRID:AB_2563812	1:200
**CD45RA**	HI100	FITC	Biolegend	304105	RRID:AB_314409	1:100
**CD45RO**	UCHL1	BV421	Biolegend	302630	RRID:AB_11126749	1:50
**CD56**	TULY56	BUV737	BD	569592	RRID:AB_3685163	1:17
**CD62L**	DREG-56	PerCP Cy5.5	Biolegend	304824	RRID:AB_2239105	1:100
**CD69**	FN50	PE-Cy5	Biolegend	310907	RRID:AB_314842	1:17
**CD8**	SK1	PE-Cy7	Biolegend	344712	RRID:AB_2044008	1:200
**Foxp3**	PCH101	PE	eBioscience	12-4776-41	RRID:AB_1518783	1:40
**Granzyme B**	GB11	PE-CF594	BD	562462	RRID:AB_2737618	1:100
**Ki67**	B56	RB670	BD	571924	RRID:AB_3686732	1:17
**PD-1**	EH12.2H7	APC	Biolegend	329908	RRID:AB_940475	1:17
**Perforin**	dG9	BV711	Biolegend	308129	RRID:AB_2687189	1:17
**TCF7/TCF1**	S33-966	RB613	BD	571352	RRID:AB_3686435	1:17
**TIM-3**	F38-2E2	BV785	Biolegend	345032	RRID:AB_2565833	1:17
**TOX**	NAN448B	RB780	BD	570193	RRID:AB_3675735	1:17

Table S3. BD Abseq antibodies

	BD AbSeq Antibody-Oligo target	Clone	Catalog number	RRID
1	CD103	2E7	940358	RRID:AB_2876229
2	CD137 (4-1BB)	1AH2	940199	RRID:AB_2876082
3	CD154 (CD40L)	MR1	940435	RRID:AB_2876290
4	CD223 (LAG3)	C9B7W	940152	RRID:AB_2876041
5	CD224.2 (2B4 B6)	2B4	940474	RRID:AB_2876320
6	CD25 (IL-2 Receptor α)	PC61	940116	RRID:AB_2876006
7	CD27	LG.3A10	940157	RRID:AB_2876046
8	CD278 (ICOS)	7E.17G9	940176	RRID:AB_2876063
9	CD279 (PD-1)	RMP1-30	940343	RRID:AB_2876215
10	CD28	37.51	940120	RRID:AB_2876010
11	CD366 (TIM-3)	5D12	940207	RRID:AB_2876089
12	CD38	90/CD38	940162	RRID:AB_2876050
13	CD4	RM4-5	940108	RRID:AB_2875998
14	CD44	IM7	940114	RRID:AB_2876004
15	CD62L (L-selectin)	MEL-14	940122	RRID:AB_2876012
16	CD69	H1.2F3	940126	RRID:AB_2876015
17	CD8a	53-6.7	940345	RRID:AB_2876217
18	KLRG1	2F1	940146	RRID:AB_2876035
19	NKG2A/C/E	20D5	940444	RRID:AB_2876298
20	TIGIT	1G9	940191	RRID:AB_2876074
