# Supplementary material for: Addition of chemotherapy to radiotherapy promotes progenitor-exhausted CD8+ T-cell clonal dominance in head and neck cancer
Source: Cancer Immunol Res. Author manuscript; Available in PMC 2026 Nov 5. (PMC7619705; doi:10.1158/2326-6066.CIR-26-0706)
Supplement: Supplementary file 9 [file EMS220945-supplement-2.pdf]

Figure S2

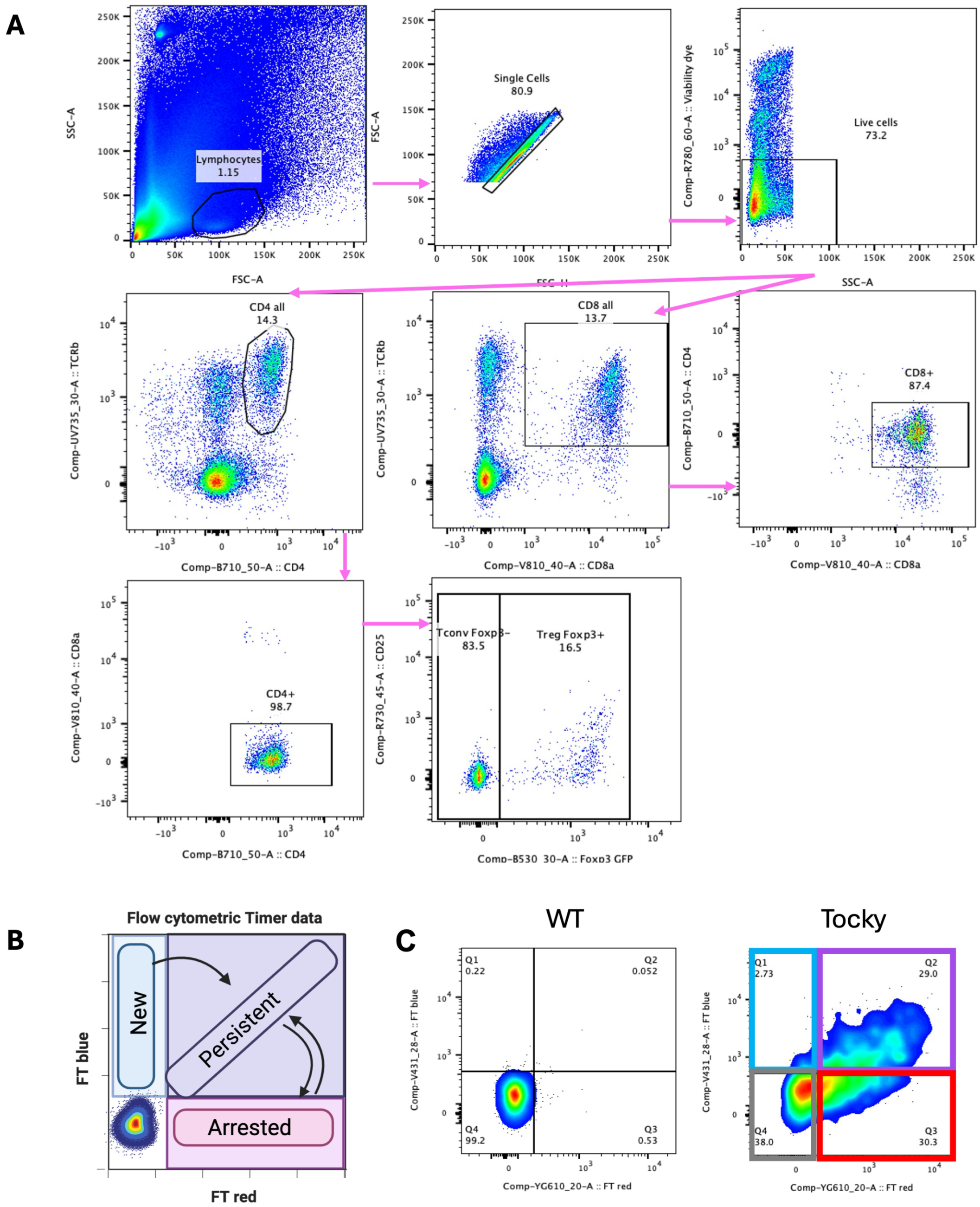

Fig. S2: Nr4a3-Tocky lymphocyte panel gating strategy.
The Nr4a3-Tocky reporter system enables *in vivo* analysis of T-cell activation dynamics via TCR engagement. Upon TCR stimulation and Nr4a3 transcription, an unstable fluorescent timer protein is initially expressed as a blue fluorophore, which matures into a red fluorophore over several hours. Newly engaged T-cells are blue-only, persistently engaged T-cells express both blue and red, arrested cells are red-only, and timer-negative cells lack both signals. **A.** All populations were gated from live single cells: conventional $CD4^+$ T-cells (Tconv), $TCR\beta^+CD4^+Foxp3^-$; regulatory T-cells (Treg), $TCR\beta^+CD4^+Foxp3^+$; $CD8^+$ T-cells, $TCR\beta^+CD8^+$. **B.** Schematic of flow cytometry timer data gating with fluorescent timer (FT) blue on the *y*-axis and FT red on the *x*-axis, classifying T-cells into “new” (FT $blue^+$ FT red^-), “persistent” (FT $blue^+$ FT red^+), “arrested” (FT $blue^-$ FT red^+), and “timer negative” (FT $blue^-$ FT red^-) populations. **C.** Example flow cytometry gating of Tocky FT populations set on the corresponding T-cell population in a wild-type (WT) sample, applied to a Tocky sample to define the “new” (blue), “persistent” (purple), “arrested” (red), and “timer negative” (grey) subsets.
