# Supplementary material for: Addition of chemotherapy to radiotherapy promotes progenitor-exhausted CD8+ T-cell clonal dominance in head and neck cancer
Source: Cancer Immunol Res. Author manuscript; Available in PMC 2026 Nov 5. (PMC7619705; doi:10.1158/2326-6066.CIR-26-0706)
Supplement: Supplementary file 10 [file EMS220945-supplement-3.pdf]

Figure S3

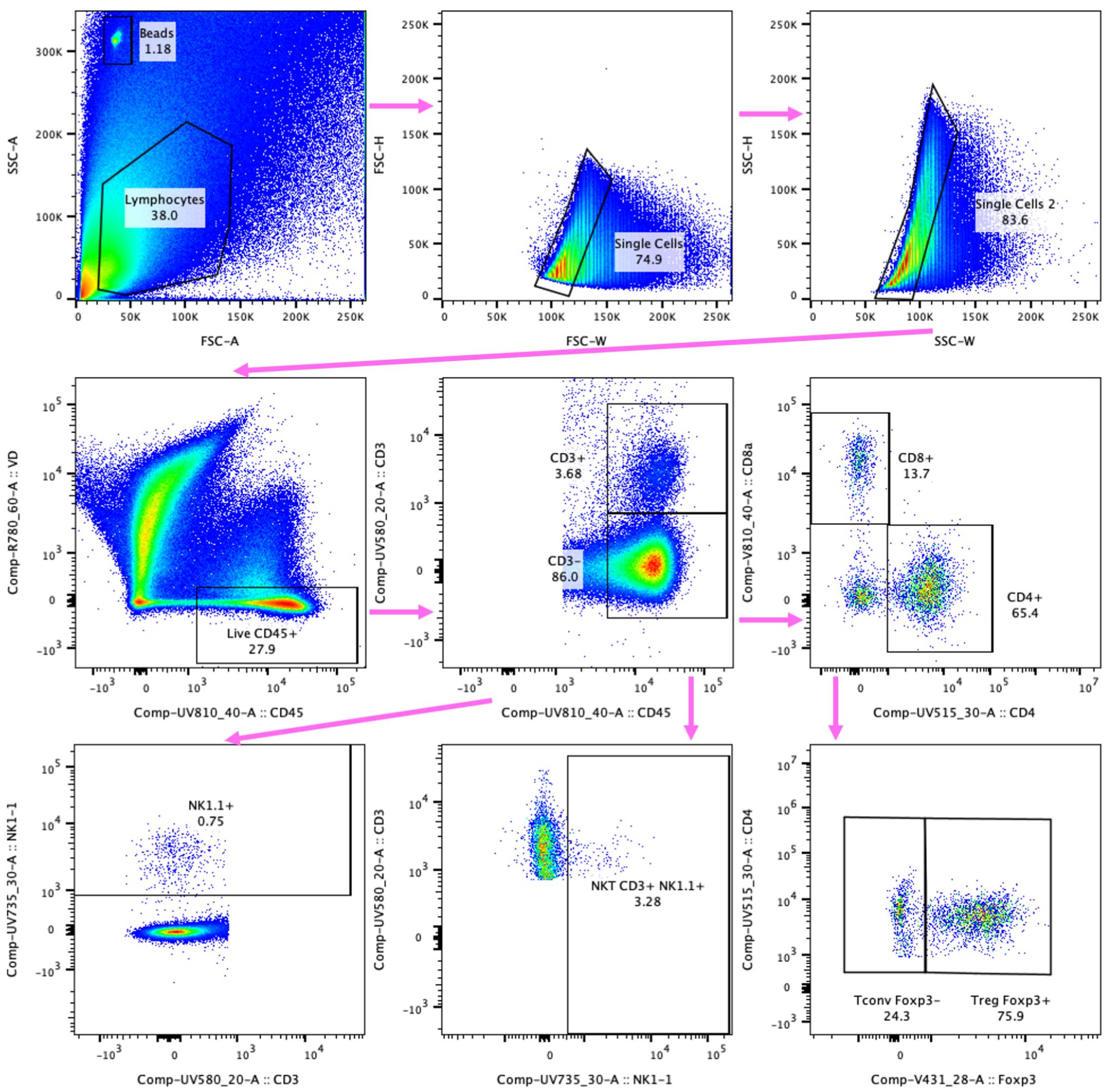

Fig. S3: Wild-type lymphocyte panel gating strategy.
Live single cells were gated for CD45⁺ expression, then classified as: Tconv, TCRβ⁺ CD4⁺ Foxp3⁻; Tregs, TCRβ⁺ CD4⁺ Foxp3⁺; and CD8⁺ T-cells, TCRβ⁺ CD8⁺.
