# Supplementary material for: Addition of chemotherapy to radiotherapy promotes progenitor-exhausted CD8+ T-cell clonal dominance in head and neck cancer
Source: Cancer Immunol Res. Author manuscript; Available in PMC 2026 Nov 5. (PMC7619705; doi:10.1158/2326-6066.CIR-26-0706)
Supplement: Supplementary file 11 [file EMS220945-supplement-4.pdf]

Figure S7

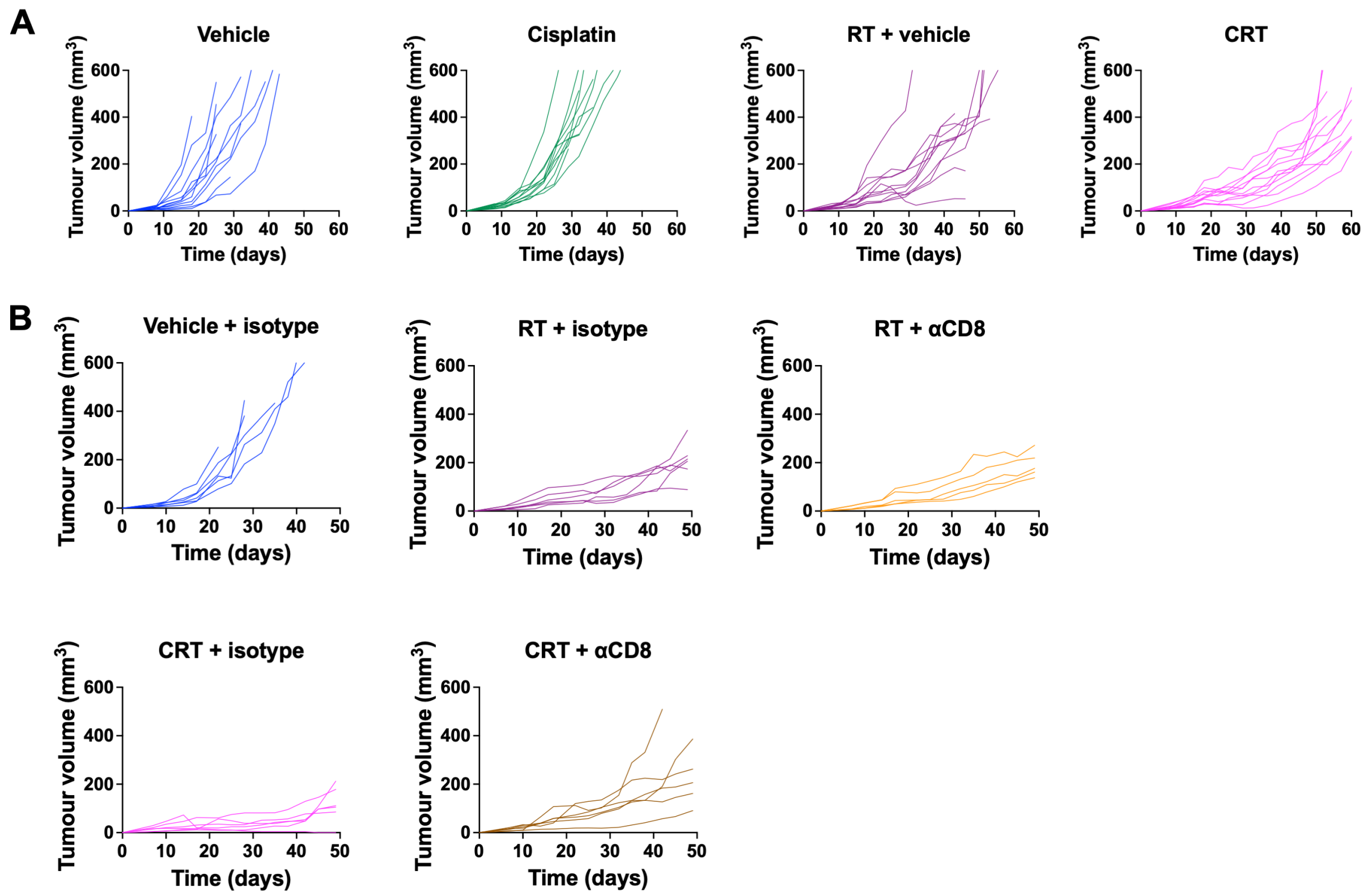

Fig. S7: Individual tumour growth curves in mEER model receiving RT and CRT.
A. CRT experiment (A-B: Vehicle n=11, Cisplatin n=11, RT + vehicle n=11, CRT n=12; 2 independent experiments). **B.** (C)RT plus $CD8^+$ T-cell depletion experiment (Vehicle + isotype n=6, RT + isotype n=6, RT + αCD8 n = 5, CRT + isotype n = 6, CRT + αCD8 n = 6; data from 1 experiment).
