# Supplementary material for: Addition of chemotherapy to radiotherapy promotes progenitor-exhausted CD8+ T-cell clonal dominance in head and neck cancer
Source: Cancer Immunol Res. Author manuscript; Available in PMC 2026 Nov 5. (PMC7619705; doi:10.1158/2326-6066.CIR-26-0706)
Supplement: Supplementary file 12 [file EMS220945-supplement-5.pdf]

Figure S4

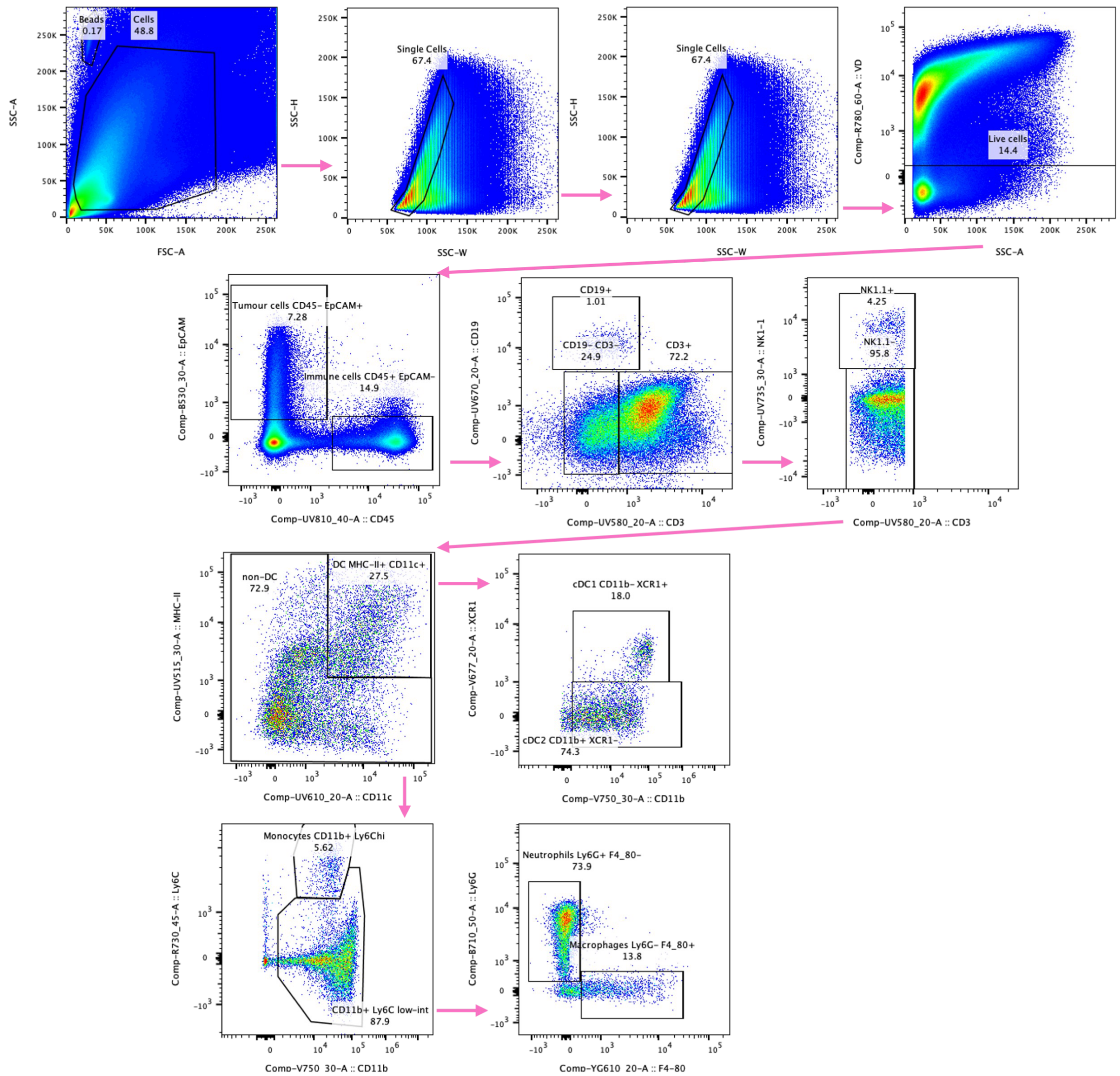

Fig. S4: Myeloid panel gating strategy.
All gated from live single cells, CD45$^+$ EpCAM$^-$ CD19$^-$ CD3$^-$. NK cells: CD3$^-$, NK1.1$^+$.DCs: NK1.1$^-$, MHC-II$^+$ CD11c$^+$; cDC1: MHC-II$^+$ CD11c$^+$ XCR1$^+$; cDC2: MHC-II$^+$ CD11c$^+$ XCR1$^-$; Neutrophils: CD11b$^+$ Ly6C$^{low-int}$ Ly6G^{high}, Monocytes: CD11b$^+$ Ly6C^{hi}, Macrophages: MHC-II$^+$ CD11b$^+$ Ly6C$^{low-int}$ F4/80$^+$; M1 Macrophages: MHC-II$^+$ CD11b$^+$ Ly6C$^{low-int}$ F4/80$^+$ CD206$^-$, M2 Macrophages: MHC-II$^+$ CD11b$^+$ Ly6C$^{low-int}$ F4/80$^+$ CD206$^+$.
