# Supplementary material for: Addition of chemotherapy to radiotherapy promotes progenitor-exhausted CD8+ T-cell clonal dominance in head and neck cancer
Source: Cancer Immunol Res. Author manuscript; Available in PMC 2026 Nov 5. (PMC7619705; doi:10.1158/2326-6066.CIR-26-0706)
Supplement: Supplementary file 13 [file EMS220945-supplement-6.pdf]

Figure S5

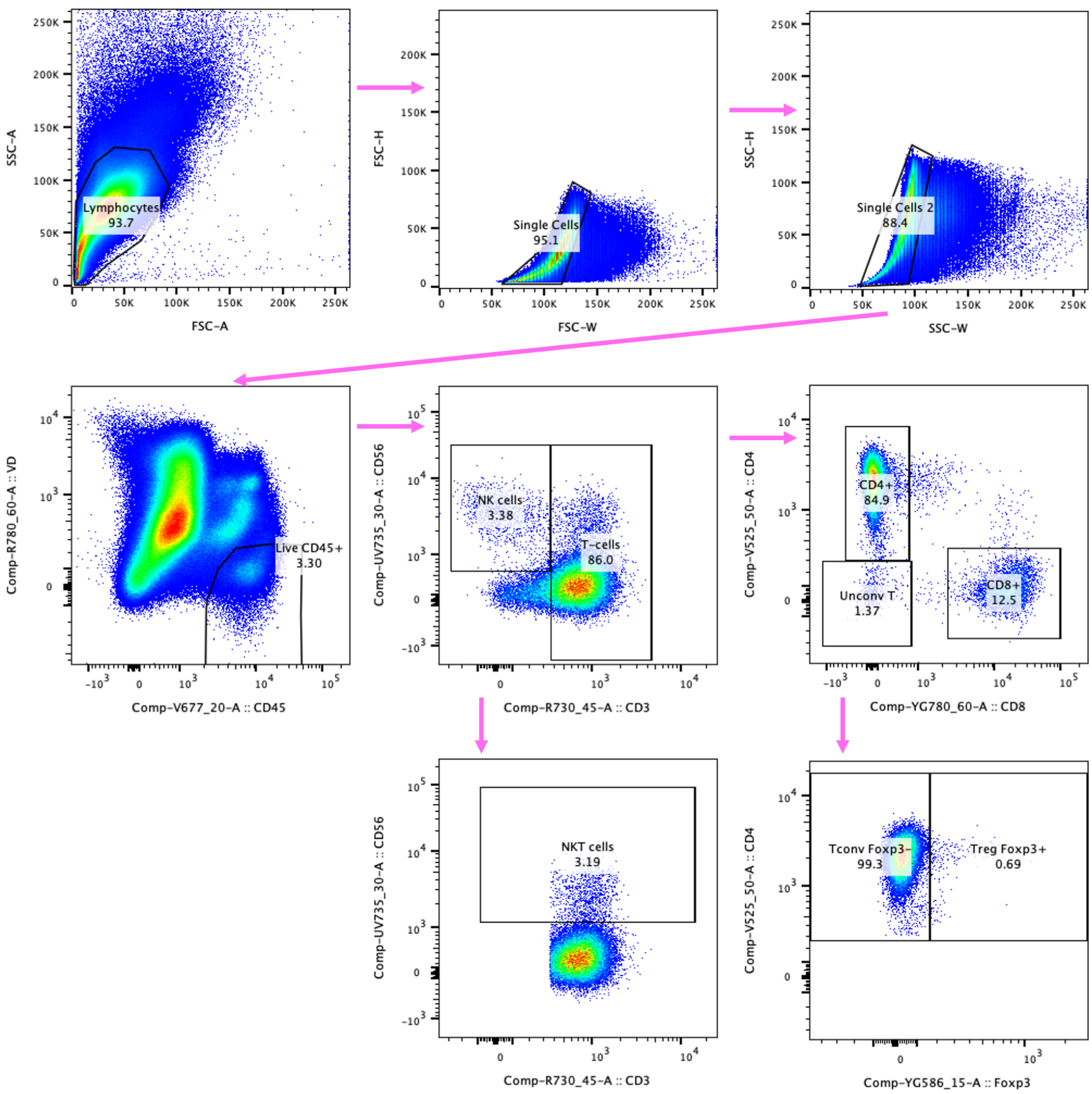

Fig. S5: Human PBMC lymphocyte gating strategy.
Live single cells were gated for $CD45^+$ expression, then classified as: NK cells, $CD3^-$ $CD56^+$; and T-cells, $CD3^+$ $CD56^-$ which were subclassified into: $CD4^+$ $Foxp3^-$; Tregs, $CD4^+$ $Foxp3^+$; and $CD8^+$ T-cells, $CD8^+$; NKT cells, $CD56^+$; and unconventional T-cells, $CD4^-$ $CD8^-$.
