# Supplementary material for: Addition of chemotherapy to radiotherapy promotes progenitor-exhausted CD8+ T-cell clonal dominance in head and neck cancer
Source: Cancer Immunol Res. Author manuscript; Available in PMC 2026 Nov 5. (PMC7619705; doi:10.1158/2326-6066.CIR-26-0706)
Supplement: Supplementary file 14 [file EMS220945-supplement-7.pdf]

Figure S6

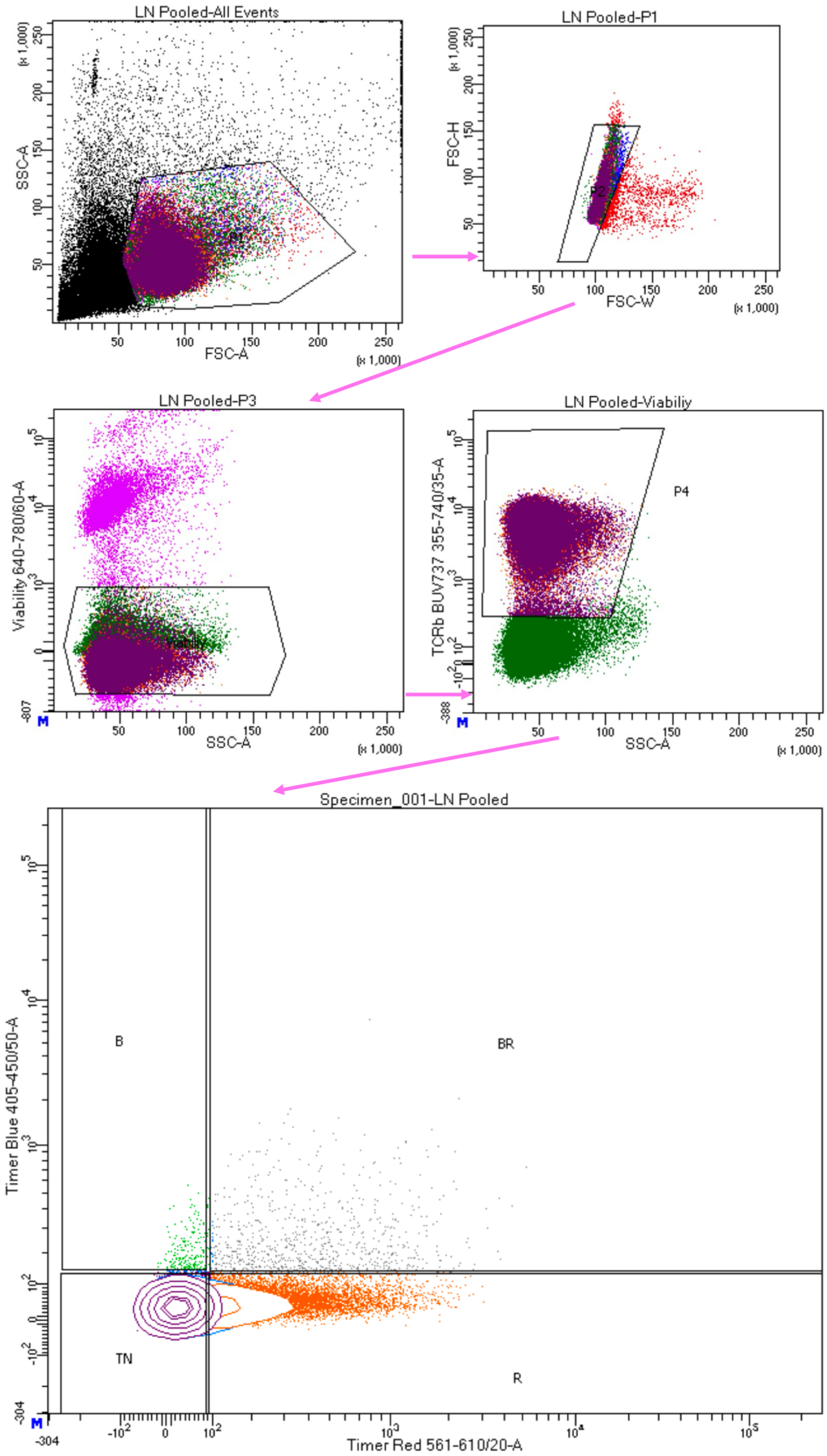

Tube: LN Pooled

Population	#Events	%Parent	%Total
All Events	215,502	####	100.0
P1	113,122	52.5	52.5
P2	111,689	98.7	51.8
P3	110,513	98.9	51.3
Viabiliy	100,000	90.5	46.4
P4	43,105	43.1	20.0
B	243	0.6	0.1
BR	806	1.9	0.4
R	12,173	28.2	5.6
TN	29,823	69.2	13.8

Fig. S6: Cell sorter gating strategy for single-cell sequencing.
Live single cells were gated for TCRβ$^+$ expression and separated into four Tocky fluorescent timer (FT) protein populations: B ("new"), FT blue$^+$ FT red$^-$; BR ("persistent"), FT blue$^+$ FT red$^+$; R ("arrested"), FT blue$^-$ FT red$^+$; and TN ("timer negative"), FT blue$^-$ FT red$^-$.
