# Supplementary material for: Addition of chemotherapy to radiotherapy promotes progenitor-exhausted CD8+ T-cell clonal dominance in head and neck cancer
Source: Cancer Immunol Res. Author manuscript; Available in PMC 2026 Nov 5. (PMC7619705; doi:10.1158/2326-6066.CIR-26-0706)
Supplement: Supplementary file 15 [file EMS220945-supplement-8.pdf]

Figure S8

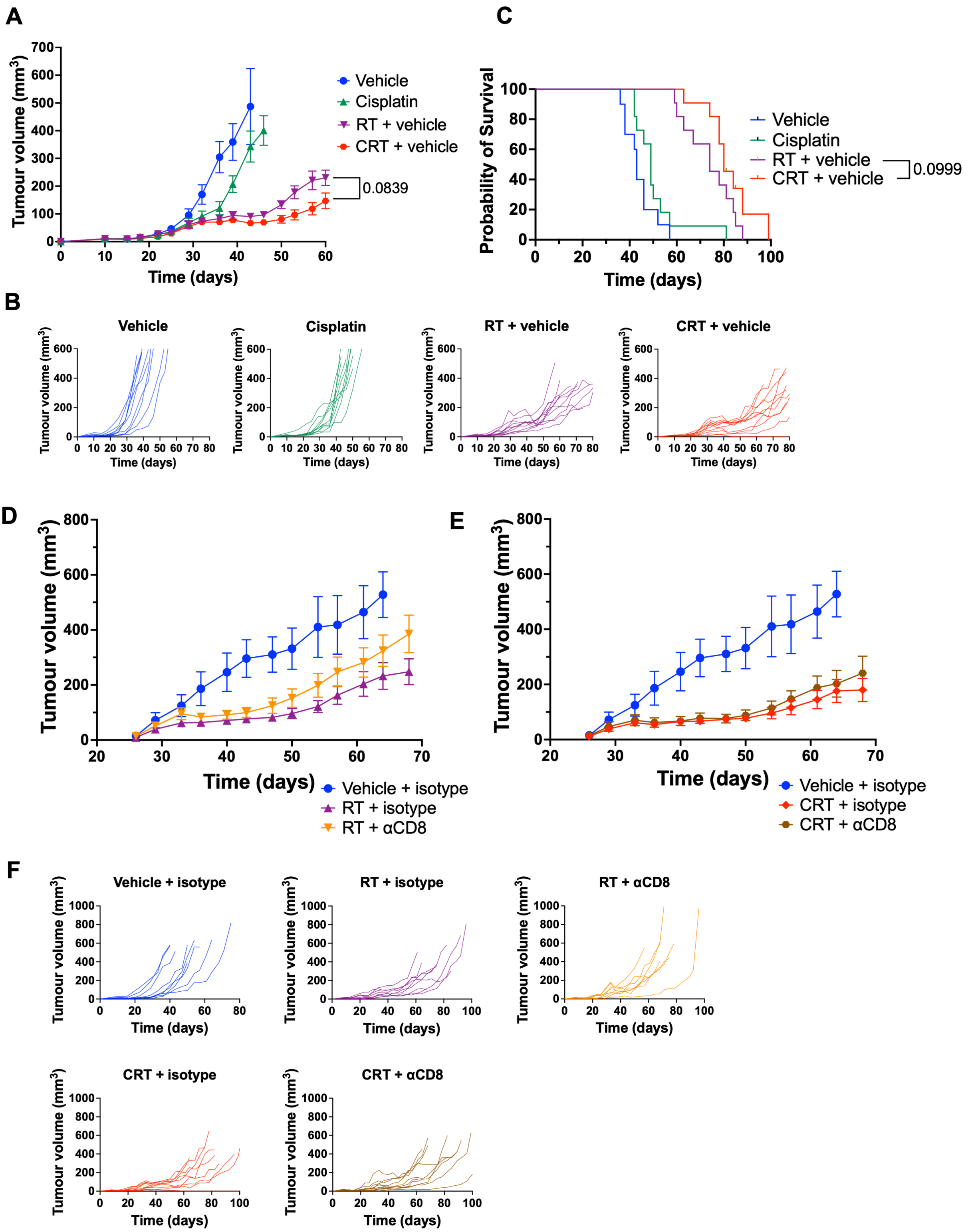

Fig. S8: (C)RT treatment efficacy and CD8⁺ T-cell dependence in the MOC1 model.
A-C: (C)RT treatment regimen in the MOC1 model included RT 6 Gy × 3 alternate days and cisplatin/vehicle 5 mg/kg intraperitoneal (i.p.) injection once prior to the first fraction of RT (Vehicle n=10, Cisplatin n=10, RT + vehicle n=11, CRT + vehicle n= 11; 2 independent experiments). **(A)** Tumour growth curves across indicated treatment conditions. **(B)** Individual tumour growth curves across indicated treatment conditions. **(C)** Survival curves across indicated treatment conditions. **D-F**: *In vivo* $CD8^+$ T-cell depletion experiments (Vehicle + isotype n=9, RT + isotype n=8, RT + αCD8 n=8, CRT + isotype n=9, CRT + αCD8 n=10; 2 independent experiments). Average tumour growth curves in mice receiving αCD8 depleting antibody/ isotype with **(D)** RT or **(E)** CRT; and **(F)** individual growth curves. Results are means ± SEM and *n* denotes mice per group.
