# Supplementary material for: Addition of chemotherapy to radiotherapy promotes progenitor-exhausted CD8+ T-cell clonal dominance in head and neck cancer
Source: Cancer Immunol Res. Author manuscript; Available in PMC 2026 Nov 5. (PMC7619705; doi:10.1158/2326-6066.CIR-26-0706)
Supplement: Supplementary file 16 [file EMS220945-supplement-9.pdf]

Figure S9

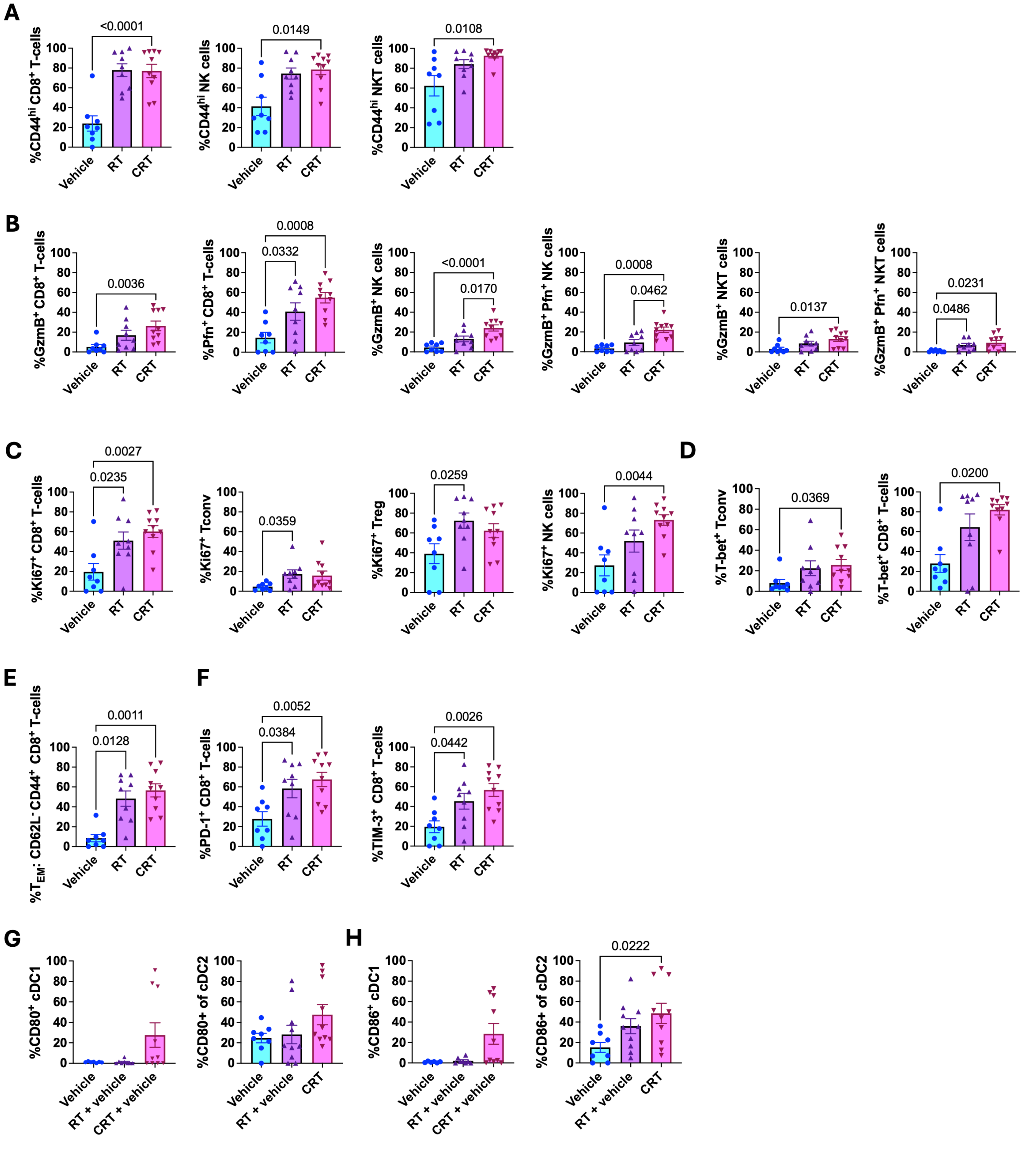

Fig. S9: Immunoprofiling via flow cytometry in mEER tumour-bearing mice.
Tumours from mice treated with vehicle, RT, or CRT were analysed by flow cytometry. **A.** Frequency (%) of $CD44^{hi}$ $CD8^{+}$ T-cells, NK cells, and NKT cells. **B.** Degranulation markers: frequency of Granzyme B^{+} (GzmB) and $Perforin^{+}$ (Pfn) $CD8^{+}$ T-cells, $GzmB^{+}$ NK cells, $GzmB^{+}Pfn^{+}$ NK cells, $GzmB^{+}$ NKT cells, and $GzmB^{+}Pfn^{+}$ NKT cells. **C.** Frequency of $Ki67^{+}$ $CD8^{+}$ T-cells, Tconv, Treg and NK cells. **D.** Frequency of $T\text{-}bet^{+}$ Tconv and $CD8^{+}$ T-cells. **E.** Frequency of effector memory (T_{em}) $CD8^{+}$ T-cells. **F.** Frequency of $PD\text{-}1^{+}$ and $TIM\text{-}3^{+}$ $CD8^{+}$ T-cells. **G.** Frequency of $CD80^{+}$ conventional type 1 dendritic cells (cDC1) and conventional type 2 dendritic cells (cDC2). **H.** Frequency of CD86+ cDC1 and cDC2. Sample sizes: vehicle n=8, RT n=9, CRT n=10; for panels G-H: n=8, n=10, n=10. Data pooled from 2 independent experiments.
